# Enhanced solid tumor cell targeting by a neoepitope-encoding oncolytic measles virus combined with CAR therapy

**DOI:** 10.1016/j.omton.2025.201043

**Published:** 2025-08-30

**Authors:** Alexander Renner, Maximiliane S.C. Finkbeiner, Ferdinand V. Haas, Anika Stahringer, Max Lindow, Nicolas Delaroque, Michael Szardenings, Stephan Fricke, Ulrike Koehl, Christine E. Engeland, Dominik Schmiedel

**Affiliations:** 1 Fraunhofer Institute for Cell Therapy and Immunology (IZI), Department for Cell and Gene Therapy Development, 04103 Leipzig, Germany; 2 Experimental Virology, Chair of Virology and Microbiology, Faculty of Health, Center for Biomedical Education and Research (ZBAF), Witten/Herdecke University, 58455 Witten, Germany; 3 Experimental Hematology and Immunotherapy, Department of Hematology, Hemostaseology, Cellular Therapy and Infectious Diseases, Faculty of Medicine and Leipzig University Hospital, University of Leipzig, 04103 Leipzig, Germany; 4 Fraunhofer Institute for Cell Therapy and Immunology (IZI), Ligand Development Unit, 04103 Leipzig, Germany; 5 Medicine Campus MEDiC of the Technical University of Dresden at Klinikum Chemnitz gGmbH, 09116 Chemnitz, Germany; 6 Institute for Clinical Immunology, University of Leipzig, 04103 Leipzig, Germany

**Keywords:** MT: Regular Issue, cancer therapy, CAR-T cell, gene therapy, oncolytic virus, CAR-NK cell, gene delivery, cell engineering, solid tumor, measles virus

## Abstract

Despite the success of cell therapy in treating hematological malignancies, the treatment of solid tumors remains challenging due to the tumor microenvironment (TME) and a lack of suitable antigens. To address this, we investigated a putative octapeptide neoepitope generated by proteolytic cleavage of the stress-induced protein MHC class I polypeptide-related sequence B (MICB). Antibodies developed via the hybridoma technique exhibited high affinity and specificity toward the octapeptide. Detection of the octapeptide was enhanced by inserting an α-helical linker before the transmembrane domain, improving accessibility on stably transduced cells. Two chimeric antigen receptor (CAR) constructs with differing single-chain variable fragment (scFv) chain orientations were expressed in primary T and natural killer (NK) cells, showing antigen-specific cytotoxicity, particularly when incorporating the rigid linker. Variations in sensitivity between CARs influenced killing efficacy and activation profiles. Oncolytic measles virus (MV) was used as a vector encoding the membrane-anchored octapeptide, selectively infecting tumor cells and enhancing CAR-T cell-mediated cytotoxicity. Combined use of CAR-T and (CAR-)NK cells demonstrated increased persistence of immune cells as well as potent and sustained antitumor effects following MV infection. This study underscores the potential of neoepitope-based CAR therapy for targeting solid tumor cells and highlights the potential synergistic effects of combining cell therapy with virotherapy for improved therapeutic outcomes.

## Graphical abstract

**Figure:**
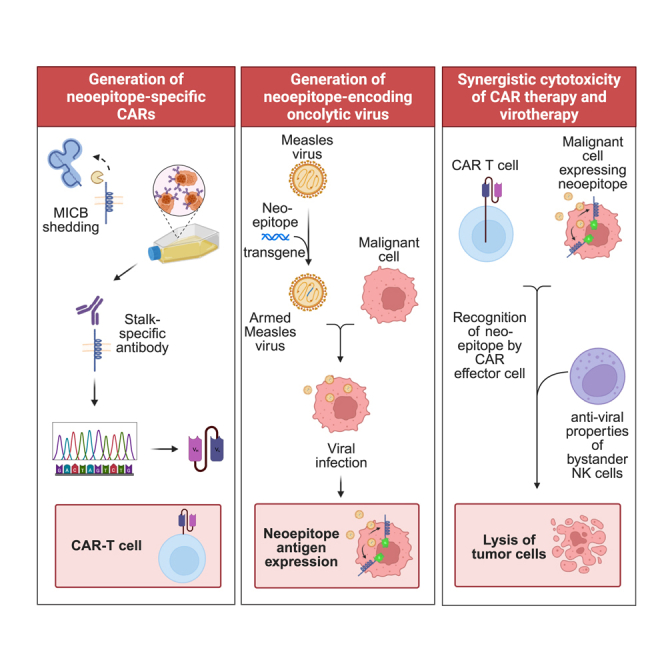

## Teaser

The study presents a novel neoepitope-based CAR strategy enabling selective targeting of solid tumors. By combining CAR-T and CAR-NK cells with oncolytic measles virus delivering the neoepitope, the approach is equipped to overcome key barriers of the tumor microenvironment, offering a promising platform for potent, specific, and sustained antitumor responses.

## Introduction

With increasing numbers of chimeric antigen receptor (CAR)-T cell therapies entering clinical trials, gaining approval, and reaching the market, this approach is rapidly changing the landscape of cancer therapy. In CAR-T cell therapy, primary T cells are genetically engineered to express a modular synthetic receptor composed of an extracellular domain, which recognizes specific tumor antigens, followed by a hinge region as well as a transmembrane and an intracellular signaling domain. This receptor enables CAR-T cells to efficiently eliminate tumor cells, a strategy that has achieved remarkable success and transformed clinical treatment regimens in hematological malignancies such as B cell lymphomas and multiple myeloma.^1^^,^^2^ However, the treatment of solid tumors remains challenging, largely due to the immunosuppressive tumor microenvironment (TME), which restricts immune cell infiltration and persistent activation as well as the limited range of targetable antigens present on the surface of tumor cells to distinguish those from healthy tissue.^3^

The TME is characterized by the presence of enzymes like matrix metalloproteinases (MMPs) and a disintegrin and metalloproteinases (ADAMs). These enzymes not only constantly modify the extracellular matrix but also have an impact on proteins expressed on cellular surfaces.^4^^,^^5^ One example of antigen shedding and subsequent immune evasion is presented by MHC class I polypeptide-related sequence A/B (MICA/B), which are stress-induced ligands often expressed in tumors. Here, shedding is facilitated by proteinases ADAM10 and ADAM17.^6^ The resulting excess soluble MICA (sMICA) in the TME can block the activating receptor NKG2D on natural killer (NK) cells, impairing their immune activity.^7^ It was shown that this suppression can be reversed by infusing high doses of NK cells with elevated NKG2D expression.^8^ Furthermore, Ferrari de Andrade et al. developed a therapeutic antibody which inhibits the shedding of MICA/B from the tumor cell surface, thereby reducing sMICA levels and restoring immune cell functionality.^9^ It is conceivable that protein remnants left on the plasma membrane after shedding may function as neoepitopes and represent promising, potentially safe targets for therapeutic intervention. While many established CAR therapies target broadly expressed proteins like CD19 or B-cell maturation antigen (BCMA), target expression often overlaps with healthy cells, posing risks for on-target off-tumor effects.^10^^,^^11^^,^^12^^,^^13^ To overcome these limitations, there is a growing need in leveraging neoantigens derived from proteins, which arise for instance due to genetic mutations or alternative processing. One way of creating safe alternatives to such established targets for CAR-T cell therapy is to find antigens primarily associated with malignant cells.

However, suitable targets alone are likely insufficient to overcome limitations of CAR therapies in solid cancers.^2^ Oncolytic virus (OV) infection of tumors has the potential to transform the TME into a more favorable site for T cell activity, potentially enhancing CAR-T cell efficacy in solid tumors. OVs are promising candidates due to their ability to preferentially infect and lyse malignant cells, releasing tumor antigens and proinflammatory signals acting as danger-associated molecular patterns (DAMPs) into the TME that stimulate immune responses.^14^^,^^15^ Due to their oncotropism, OVs represent potent antitumoral agents as well as vectors for the delivery of therapeutic antigens. OVs were previously shown to successfully deliver therapeutic transgenes like cytokines, immune checkpoint inhibitors, and tumor suppressors as well as cell-cell adhesion proteins to be expressed on the cell surface.^16^^,^^17^^,^^18^^,^^19^^,^^20^ One safe and adaptable platform is the attenuated, replication-competent Edmonston B measles vaccine virus, which preferentially targets transformed cells by exploiting the overexpression of CD46 in many malignant cells.^21^^,^^22^^,^^23^^,^^24^ In preclinical studies and early clinical trials, combining CAR-T cells with OVs has shown the potential to increase tumor infiltration, enhance antigen presentation, and mitigate T cell exhaustion.^25^^,^^26^ Due to the range of tumor heterogeneity, highly permissive tumor cells are expected to be killed by OVs, while cells defying oncolysis, which still express viral transgenes, could be targeted by CAR-T cells.^27^^,^^28^^,^^29^^,^^30^ Therefore, further enhancements for CAR-T cell therapy can be realized with combination approaches, which are emerging as powerful strategies that offer the potential to overcome existing challenges and expand therapeutic possibilities.

We hypothesized that using a protein-derived neoepitope primarily upregulated in transformed cells experiencing stress and contributing to tumor escape mechanisms will be favorable for the efficacy of CAR therapy, since surface expression of MICA/B is typically low or absent in healthy tissues.^31^ Based on this hypothesis, we generated antibodies against an octapeptide neoepitope designed along the lines of physiological cleavage patterns of the MICB protein. Furthermore, combining the antitumoral effect of CAR therapy with virus-mediated cytolytic and immunostimulatory effects of transgene-encoding measles virus (MV) may boost potency. Therefore, we used one antibody to generate CAR molecules expressed in T and NK cells targeting the octapeptide for evaluation of cytotoxicity toward transduced tumor cells. Finally, we introduced MV as a vector encoding for the membrane-anchored octapeptide fused to an intracellular EGFP, aiming to combine the antitumoral CAR effect with the oncolytic features of MV to synergistically kill malignant cells, which may be beneficial particularly for the treatment of solid tumors.

## Results

### An MICB-derived octapeptide was used to successfully generate two murine antibodies with high antigen affinity and specificity

**Figure 1:**
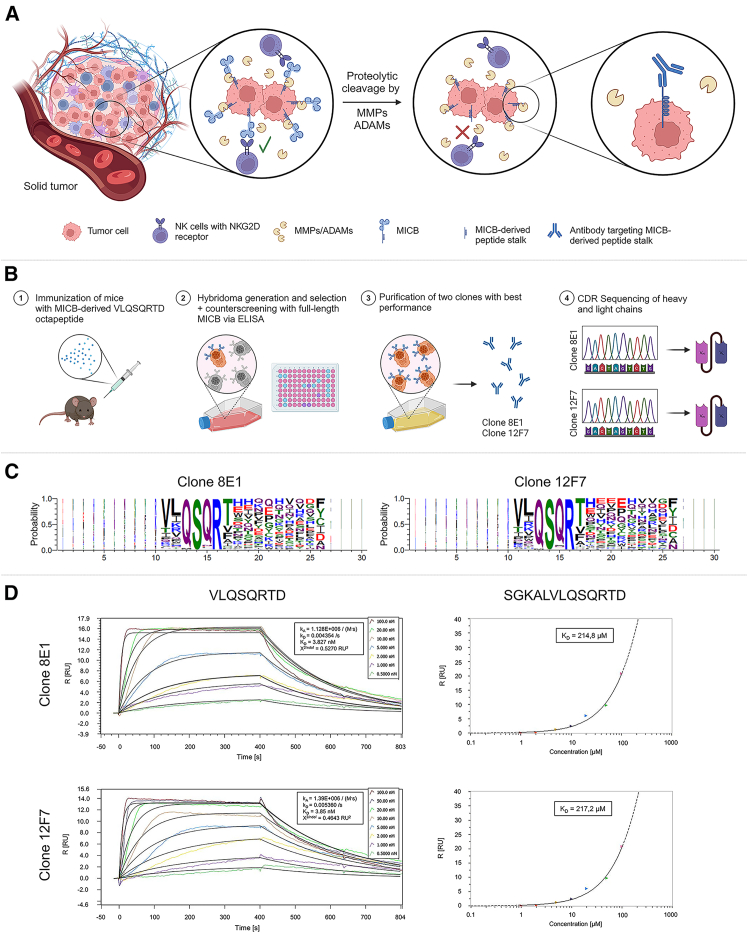
Antibodies generated against an MICB-derived octapeptide show high affinity to the immunization sequence (A) Illustration of proteolytic shedding of MICB by MMPs and ADAMs from the tumor cell surface leading to failed recognition by immune cells like natural killer cells via innate receptors. Antibodies can be raised specifically against the peptide stalk remaining on the cellular surface and can be used for therapeutic purposes. (B) Schematic of the application of the hybridoma technique for generating antibodies. (1) Mice were immunized with the VLQSQRTD-keyhole limpet hemocyanin (KLH) peptide conjugate. (2) Hybridomas were generated and selected via hypoxanthine-aminopterin-thymidine (HAT) medium. Screening was carried out using ELISA including counter screening against full-length MICB. (3) Clones 8E1 and 12F7 were purified, and (4) complementarity-determining region (CDR) sequencing was performed. (C) Epitope mapping results: the weblogo was generated by aligning those sequences from the experiments with antibodies 8E1 and 12F7 that had been found at least 100 times and comprised at least one of the 4-mer motifs VLQS, LQSQ, QSQR, SQRT, and QRTD. (D) Sensorgrams for determination of K_D_ values of monoclonal antibody (mAb) 8E1 and 12F7 with the octapeptide VLQSQRTD (top row) and the N-terminally elongated peptide SGKALVLQSQRTD (bottom row) using surface plasmon resonance (SPR). Single-cycle kinetics analysis was carried out by monitoring the transitions of immobilized monoclonal antibodies (mAbs) after addition of increasing concentrations (0.5 nM–100 nM) of VLQSQRTD or SGKALVLQSQRTD (1 μM–200 μM).

To our knowledge, no antibodies targeting the peptide stalk of MICB remaining on the cellular surface after shedding have been described. Therefore, we assessed the potential of such a neoepitope for therapeutic purposes (Figure 1A). Based on data published by Waldhauer and colleagues,^6^ we designed an octapeptide known to be one of the products of proteolytically cleaved MICA/B by MMPs and ADAMs, terminating N-terminally of the annotated transmembrane domain of MICB. This octapeptide with the amino acid sequence VLQSQRTD was then used for immunization of mice to generate murine antibodies using the hybridoma technique (Figure 1B). During the screening process, counter screenings with full-length MICB were performed via ELISA to avoid cross-reactivity. Strongest binders within polyclones were chosen based on ELISA screening and were further validated by screening the affinity of antibodies in hybridoma supernatants toward A375 cells stably transduced to express the peptide VLQSQRTD on their surface. In addition to the octapeptide, an N-terminally elongated peptide of 13 amino acids, SGKALVLQSQRTD,^6^ also known to be a potential product of MICB shedding, was used during the screening of hybridoma supernatants on transduced A375 cells. After a final round of subclone screening, two monoclonal antibodies, clones 8E1 and 12F7, were identified as the most promising candidates and were purified. Complementarity-determining region (CDR) sequences of both antibodies were retrieved from cDNA generated from hybridoma cells by sequencing.

For a more in-depth characterization of the binding properties of both antibodies, epitope mapping was carried out using a phage display library to determine the precise amino acids required for binding. Screening the peptide library against antibodies 8E1 and 12F7 revealed enriched peptide motifs that provided insight into epitope recognition. Both antibodies displayed overlapping core sequences, suggesting they target the same epitope within the MICB-derived octapeptide (Figure 1C). The 4-mer QSQR was identified as the most abundant consensus sequence within the octapeptide VLQSQRTD, with the N-terminal valine and the threonine at position seven also representing highly abundant amino acids in about 50% of enriched peptides. Additionally, N-terminally of the valine, a clear-cut without preference for any specific amino acid, was identified.

For affinity measurements, surface plasmon resonance (SPR) analysis was performed to characterize the binding kinetics of both antibodies to the soluble octapeptide VLQSQRTD as well as the N-terminally elongated peptide SGKALVLQSQRTD. The analysis revealed high antibody-antigen affinities with K_D_ values of 3.83 and 3.85 nM for antibodies 8E1 and 12F7, respectively (Figure 1D), indicating strong and specific binding. K_D_ values for interaction with the longer peptide were considerably higher with 214.8 and 217.2 μM, respectively.

### Connecting the octapeptide to a rigid linker significantly enhances its detectability on the cell surface

**Figure 2:**
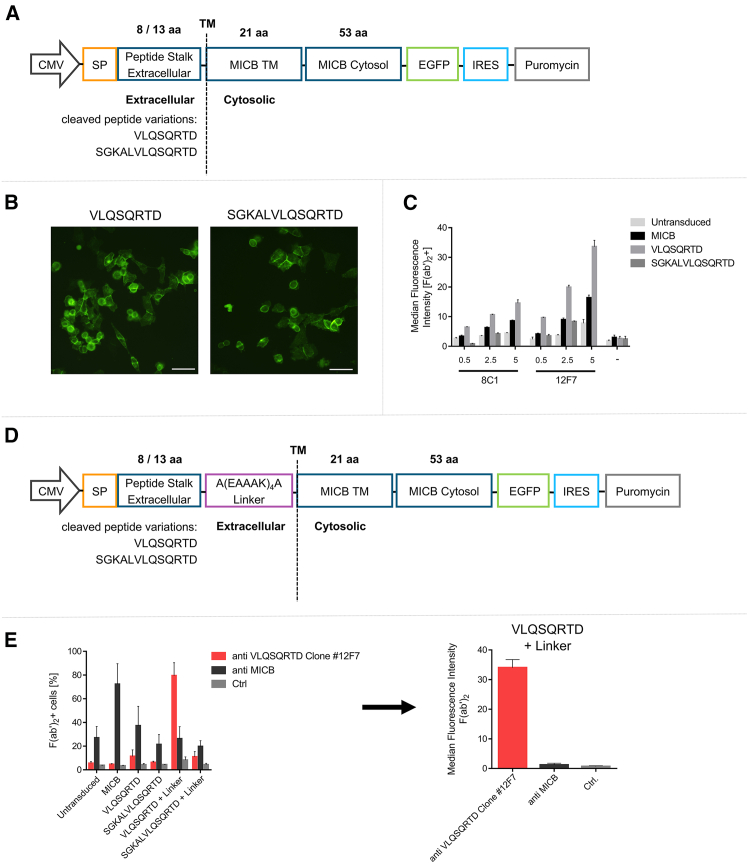
Epitope recognition by octapeptide-targeting antibodies is enhanced through insertion of a rigid linker (A) Schematic representation of the expression cassette used to mimic enzymatic shedding of MICB, leaving either the octapeptide VLQSQRTD or the 13 amino acid-long peptide SGKALVLQSQRTD on the cellular surface. (B) Representative images of A375 cells expressing the VLQSQRTD-EGFP fusion protein and the SGKALVLQSQRTD-EGFP fusion protein on their cellular surface. Green fluorescence depicts EGFP expression localized at the membrane. Scale bar represents 50 μm. (C) Median fluorescence intensity (MFI) from anti-mouse F(ab’)_2_-stained A375 cells after detection with increasing concentrations of the two specifically raised murine antibodies 8E1 and 12F7. Data are shown from *n* = 3 biological replicates as mean (SEM). (D) Schematic representation of the adapted expression cassette used to express either VLQSQRTD or SGKALVLQSQRTD with the inclusion of a rigid (EAAAK)_4_A linker between the extracellular and transmembrane domains on the cellular surface. (E) Left plot depicts anti-mouse F(ab’)_2_ signal of stably transduced Mac-1 cells with the indicated transgenes after incubation with antibody 12F7. Right plot shows MFI of anti-mouse F(ab’)_2_-stained Mac-1 VLQSQRTD-Linker transduced cells after incubation with clone 12F7. Data from *n* = 3 are depicted as mean (SEM).

After characterizing the binding properties of antibody clones 8E1 and 12F7 to the antigen peptide VLQSQRTD in solution, their binding was further assessed in a cellular context to confirm recognition in practical applications. For that purpose, an expression cassette was designed to mimic the cleavage product remaining on the cellular surface after enzymatic shedding of MICB resulting in the exposure of the octapeptide (Figure 2A). To verify adequate expression prior to assessing antibody binding, A375 cells were transduced with lentiviral vectors (LVs) to stably express either the immunization peptide VLQSQRTD or the N-terminally elongated version of the peptide SGKALVLQSQRTD. A375 cells were analyzed microscopically to determine the localization of the MICB-derived peptide-EGFP fusion protein within the cell. EGFP signals could be localized primarily to the plasma membrane, confirming the correct expression of the protein and functional localization of the transmembrane domain in the cellular membrane despite the shortened extracellular peptide (Figure 2B). Based on these findings, both clones 8E1 and 12F7 were employed to detect the peptide on the cell surface. Increasing the antibody concentrations from 0.5 to 5 μg resulted in higher median fluorescence intensities (MFIs). However, it also led to increased nonspecific binding in samples with excess antibody, even in the absence of VLQSQRTD expression (Figure 2C). Overall, higher MFIs were measured using clone 12F7 compared to clone 8E1.

Presumably, the limited specific detection on A375 cells is caused by steric hindrance through surrounding proteins on the cellular surface, obstructing antibody binding to the peptide sequence which is in close proximity to the membrane. Therefore, an adapted cassette for the expression of the peptides was created, first as a proof of concept, to include a rigid (EAAAK)_4_A linker between the extracellular peptide and the transmembrane domain of MICB (Figure 2D). These adapted expression cassettes were then used to generate Mac-1 cells stably expressing the peptides with an additional linker in the extracellular domain. Using 1 μg of clone 12F7, the octapeptide VLQSQRTD without the linker was detected on approximately 12% of cells (Figure 2E), while controls—such as untransduced cells or cells transduced with full-length MICB or SGKALVLQSQRTD—showed minimal signal. Through a more membrane-distal expression of the octapeptide achieved by the insertion of the rigid linker, around 80% of positively stained cells could be detected, resulting in an MFI amply exceeding nonspecific staining (Figure 2E). Additionally, on U-87 MG and MeWo cells transduced to stably express the peptides including the linker, 98% and 99% of cells expressing the VLQSQRTD peptide stained positive, compared to almost no detection when transduced with the SGKALVLQSQRTD peptide (Figure S1).

### CAR-T cells directed against the octapeptide VLQSQRTD display target-dependent cytotoxicity

**Figure 3:**
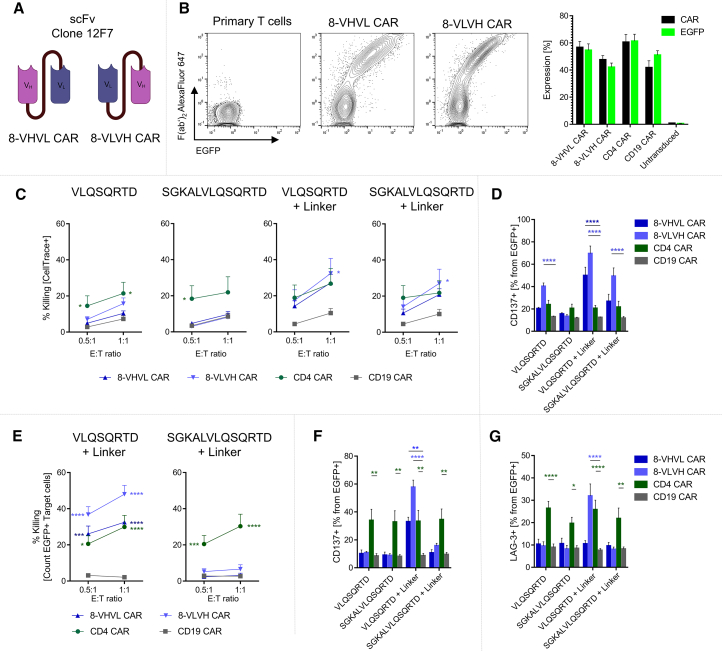
CAR-T cells targeting the MICB-derived octapeptide show CAR-dependent activation and efficiently kill tumor cells (A) Schematic representation of scFv of both 8-VHVL CAR and 8-VLVH CAR targeting the octapeptide VLQSQRTD used in this study. (B) Expression of CAR molecules on T cells. Left panel shows representative plots of flow cytometry data of primary T cells, 8-VHVL CAR-T and 8-VLVH CAR-T. Right panel shows expression of both experimental CARs (8-VHVL and 8-VLVH) as well as CD4 and CD19 CAR on T cells. Black bars represent CAR expression detected via anti-mouse F(abʹ)_2_ antibody, and green bars represent EGFP signal; data from *n* = 18 independent experiments are depicted as mean (SEM). (C) CAR-T cells were cocultured with Mac-1 cells stably transduced to express indicated transgenes. Tumor cells were labeled with CellTrace Violet, and killing was assessed via PI staining. Killing is depicted as ratio of PI+ cells in cocultures and independently cultured Mac-1 cells. Data from *n* ≥ 6 experiments are depicted as mean (SEM). (D) CD137 expression was measured on CAR-T cells after incubation with Mac-1 for 24 h. Data are shown from EGFP+ CAR-T cells and are depicted from *n* = 6 independent experiments as mean (SEM). (E) CAR-T cells were cocultured with T cells from the same donor transduced to express indicated transgenes. T cells used as target cells were labeled with CellTrace Violet, and killing was assessed via PI staining. Only EGFP+ target T cells were analyzed for PI staining. Killing is depicted as the ratio of PI+ cells in cocultures and independently cultured target T cells. Data from *n* = 9 independent experiments are shown as mean (SEM). (F) CD137 expression was measured on CAR-T cells after incubation with transduced T cells as target cells for 24 h. Data are shown from EGFP+ CAR-T cells and are depicted from *n* = 6 independent experiments as mean (SEM). (G) LAG-3 expression was measured on CAR-T cells after incubation with transduced T cells as target cells for 72 h. Data are shown from EGFP+ CAR-T cells from *n* = 4 experiments as mean (SEM); statistical analysis of (C) through (G) was performed by two-way ANOVA followed by Dunnett’s multiple-comparisons test, and experimental groups were compared to CD19 CAR. ∗ = *p* ≤ 0.05, ∗∗ = *p* ≤ 0.01, ∗∗∗ = *p* ≤ 0.001, and ∗∗∗∗ = *p* ≤ 0.0001.

Building on the successful characterization of both antibody clones derived from hybridomas and the design of the peptide neoepitope, including the potential incorporation of a rigid linker between the extracellular peptide and the transmembrane (TM) domain of MICB, we aimed to engineer a CAR specifically targeting the octapeptide VLQSQRTD. Variable heavy (V_H_) and variable light (V_L_) chains were obtained from hybridoma cells by sequencing both antibody clones. Comparison of both clones revealed only minor variations in nucleotide sequences ultimately leading to very similar proteins; therefore, only clone 12F7 was chosen to serve as the basis for CAR generation. Two variants of the CAR were created by varying the order of V_H_ and V_L_ in the single-chain variable fragment (scFv) domain of the molecule (Figure 3A), which are referred to as 8-VHVL CAR and 8-VLVH CAR, respectively. Expression of both CARs on primary T cells using a gammaretroviral vector was successfully shown with mean expression values of 56.7% and 47.6%, respectively (Figure 3B).

To study CAR-derived cytotoxicity, we cocultured CAR-T cells with Mac-1 cells stably transduced to express either VLQSQRTD or SGKALVLQSQRTD with or without a rigid linker on the cell surface. Mac-1 cells were labeled with the fluorescent dye CellTrace Violet and were cultured in effector-to-target (E:T) ratios of 0.5:1 and 1:1 (Figure 3C). Cell death of Mac-1 cells was assessed after 16 h using propidium iodide (PI) staining. This approach allowed us to quantify the reduction in live Mac-1 cells caused by CAR-T cell activity. CAR-T cells directed against CD4 were used as a positive control due to Mac-1 cells naturally expressing CD4. Killing induced by those CD4-specific CAR-T cells was comparable across all Mac-1 transfectants. On Mac-1 cells expressing the membrane-anchored octapeptide without a linker, 8-VLVH CAR-T cells demonstrated measurable cytotoxic activity, achieving 18.1% killing at an E:T ratio of 1:1. In contrast, 8-VHVL CAR-T cells showed only minimal cytotoxicity under the same conditions. Additionally, no cytotoxic activity was observed on Mac-1 cells expressing the 13-amino acid peptide SGKALVLQSQRTD without a linker, mirroring previous results from characterizing clone 12F7, which this CAR is based on. Inserting the rigid linker significantly enhanced cytotoxicity for both 8-VHVL CAR-T and 8-VLVH CAR-T cells, achieving killing rates of up to 36.6%. Similarly, equipping Mac-1 cells with SGKALVLQSQRTD + Linker increased killing efficiency, although to a lesser extent compared to the shorter peptide VLQSQRTD. Coculture of CAR-T cells with Mac-1 for an extended period of 72 h enhanced cytotoxicity up to 76.1% and 40.4% for 8-VLVH and 8-VHVL CAR, respectively (Figure S2A). Additionally, CD137 (4-1BB) expression on 8-VHVL and 8-VLVH CAR-T cells cocultured with Mac-1 VLQSQRTD + Linker for 24 h was shown to be significantly higher compared to CD19 CAR-T cells, with 8-VLVH CAR-T cells showing the highest expression. Coculture with Mac-1 SGKALVLQSQRTD + Linker also induced an increase in CD137 expression in 8-VLVH CAR-T cells, however, not in 8-VHVL CAR-T (Figure 3D). Following coculture with Mac-1 VLQSQRTD, significantly higher CD137 levels were observed on 8-VLVH CAR-T cells in comparison to CD19 CAR-T cells.

To assess if target cells with low antigen expression can effectively be lysed by the newly created CARs, we transduced primary T cells with the peptides incorporating the extracellular linker. Then, we set up cocultures with CAR-T cells from matching donors to minimize variability by ensuring genetic uniformity. Here, we measured cell death of T cells transduced with the target antigens after 16 h using PI staining. We observed specific killing of T cells expressing VLQSQRTD + Linker using both configurations of the CAR, with 8-VLVH CAR showing elevated killing compared to 8-VHVL CAR, which was comparable to CD4 CAR-T (Figure 3E). Expression of SGKALVLQSQRTD + Linker on T cells did lead to slight, but insignificant, elevation of killing elicited by 8-VLVH CAR-T. Elongated coculture for an extended period of 72 h elevated target cell killing to 73.2% and 57.8% for 8-VLVH and 8-VHVL CAR, respectively (Figure S2B). Analyzing CD137 expression on 8-VHVL and 8-VLVH CAR-T cells after 24 h of coculture, we observed T cell activation with 8-VLVH CAR-T cells displaying increased expression over 8-VHVL CAR-T cells, which is in line with observations made from cytotoxicity assays conducted simultaneously (Figure 3F). To evaluate cellular exhaustion on CAR-T cells after a prolonged co-incubation period of 72 h, LAG-3, TIM-3, and PD-1 were stained on CAR-T cells from cocultures. Here, we observed a strong increase in LAG-3 expression on 8-VLVH CAR-T cells cultured with T cells transduced to express VLQSQRTD + Linker, exceeding LAG-3 expression of CD4 CAR-T (Figure 3G), indicating a strong cellular activation. Similarly, levels of TIM-3 were also increased in 8-VLVH CAR-T cells upon coculture with T cells expressing VLQSQRTD + Linker, while PD-1 was not induced on the peptide-specific CARs in coculture with the different target cells (Figure S3).

### Membrane-anchored octapeptide expression on tumor cells mediated by MV enables cytotoxicity by CAR-T cell therapy

**Figure 4:**
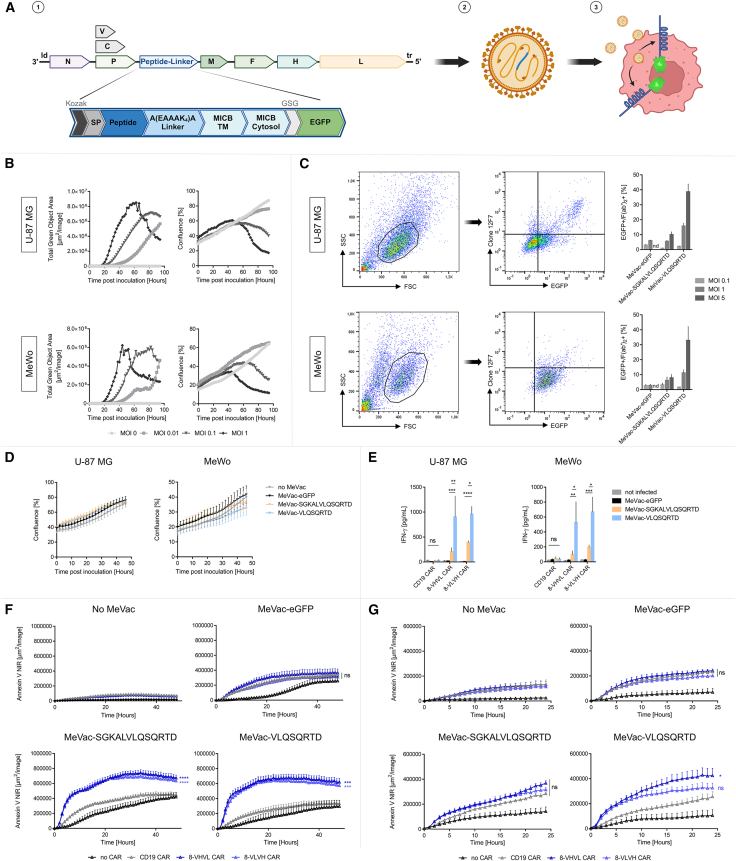
Combination of OV and CAR-T cell therapy specifically eliminates tumor cells (A) Illustration of recombinant MeVac-VLQSQRTD and MeVac-SGKALVLQSQRTD. (1) The transgene is encoded downstream of the P open reading frame within the MV genome. Kozak and MICB leader sequences mediate efficient translation and expression. (2) Transgene-encoding viruses are rescued through a reverse genetics system. (3) Peptide linkers including intracellular EGFP get expressed upon infection. (B) U-87 MG and MeWo cells were titrated with MeVac-EGFP ranging from MOI 0.01 to 1 and incubated for 96 h. Respective representative plots on the left depict EGFP signal, while representative plots on the right show confluence of U-87 MG and MeWo, respectively. (C) U-87 MG and MeWo cells were incubated for 48 h after inoculation with different MOIs of MeVac-eGFP, MeVac-SGKALVLQSQRTD, or MeVac-VLQSQRTD. Inoculated cells were stained for expression of VLQSQRTD with clone #12F7 and a secondary anti-mouse F(abʹ)_2_ antibody. Gating on U-87 MG and MeWo is shown in the left panel from one exemplary experiment using an MOI of 1 of MeVac-VLQSQRTD. Right panel shows EGFP+/F(abʹ)_2_+ cells after staining. Values from *n* = 4 independent experiments are depicted as mean (SEM). (D) U-87 MG (left) and MeWo (right) were inoculated with an MOI of 0.1 for MeVac-eGFP and an MOI of 1 for both MeVac-SGKALVLQSQRTD and MeVac-VLQSQRTD and incubated for 48 h. The confluence of cells prior to addition of effector cells is depicted from *n* = 4 independently conducted experiments as mean (SEM). (E) Supernatant of CAR-T- and MV-treated tumor cells was harvested 72 h after addition of effector cells. IFN-γ levels were measured via ELISA. Data from *n* = 4 experiments are shown as mean (SEM). (F) U-87 MG cells were inoculated with MeVac-eGFP, MeVac-SGKALVLQSQRTD, or MeVac-VLQSQRTD. Forty-eight h after inoculation, CAR-T cells were added and apoptosis was monitored by addition of Annexin V NIR dye to the culture medium. Data from *n* = 6 independent experiments are shown as mean (SEM). (G) MeWo cells were inoculated with MeVac-eGFP, MeVac-SGKALVLQSQRTD, or MeVac-VLQSQRTD. Forty-eight h after inoculation, CAR-T cells were added and apoptosis was monitored by addition of Annexin V NIR dye to the culture medium. Data from *n* = 3 independent experiments are shown as mean (SEM); statistical analysis of (E) was performed by two-way ANOVA with Tukey’s multiple-comparisons test; statistical analysis of (F) and (G) was performed by two-way repeated measures ANOVA with Tukey’s multiple-comparisons test. ∗ = *p* ≤ 0.05, ∗∗ = *p* ≤ 0.01, ∗∗∗ = *p* ≤ 0.001, and ∗∗∗∗ = *p* ≤ 0.0001.

Combining engineered oncolytic viral vectors with CAR-T cell therapy may be able to tackle challenges connected to treating solid tumors such as the immunosuppressive TME and lowered tumor infiltration as well as antigen heterogeneity. Having demonstrated that CAR-T cells can specifically target the VLQSQRTD peptide when fused to the TM and intracellular domains of MICB with a rigid linker on transduced cells in cytotoxicity assays, we next focused on harnessing MV as a vector to selectively infect tumor cells and drive transgene expression. To achieve this, we engineered live-attenuated MV Schwarz vaccine strain vectors to encode the same peptide-EGFP fusion protein with a rigid linker in an additional transcription unit as displayed in Figure 2D, resulting in MeVac-VLQSQRTD and MeVac-SGKALVLQSQRTD (Figure 4A). To characterize the response of our model cell lines to MV, we first inoculated the glioblastoma cell line U-87 MG and the melanoma cell line MeWo for 2 hours with MV vector carrying a GFP transgene, named MeVac-eGFP, which was first described by Veinalde et al.^32^ All viral strains showed functional replication and similar growth rates in both cell lines (Figure S4). The increase in total green object area as well as the confluence of the cell culture was then monitored for 4 days. Compared to non-inoculated cells, both cell lines showed a multiplicity of infection (MOI)-dependent increase of GFP (Figure 4B). Cytopathic effects started to set in concurrently with peaking GFP expression in the cultures and confluence progressively declined.

To assess the ability of MeVac-VLQSQRTD to drive the expression of the peptide-EGFP fusion protein following tumor cell infection, U-87 MG and MeWo were cultured for 48 h post-inoculation, and expression was analyzed using antibody clone 12F7. The highest peptide expression occurred at an MOI of 5. However, this high titer also caused substantial cytopathic effects in the tumor cells. At an MOI of 1, VLQSQRTD peptide expression was detected on approximately 20% of U-87 MG and 10% of MeWo, with noticeably reduced cytopathic effects (Figure 4C). In contrast, no distinct expression above nonspecific staining was detected at an MOI of 0.1. For the elongated peptide SGKALVLQSQRTD, detectable expression was considerably lower at MOIs of 1 and 5. To limit cytopathic effects during early infection stages, an MOI of 1 was selected for both MeVac-VLQSQRTD and MeVac-SGKALVLQSQRTD in subsequent experiments. In comparison, MeVac-eGFP was used at an MOI of 0.1, as its higher cytopathic effects at MOI 1 were evident (Figure 4B). These optimized MOIs allowed regular tumor cell growth prior to the addition of effector cells (Figure 4D). Building on these results and the confirmed ability of MV to mediate expression of peptide-linker constructs on tumor cells, we proceeded to evaluate CAR-T cell responses.

CAR-T cells were applied to MV-infected tumor cells 48 h after initial inoculation. MeVac-VLQSQRTD infection of both U-87 MG and MeWo caused significantly higher interferon (IFN)-γ levels compared to MeVac-eGFP and MeVac-SGKALVLQSQRTD in cocultures with both 8-VHVL and 8-VLVH CAR, however, not CD19 CAR (Figure 4E). Notably, infection with MeVac-SGKALVLQSQRTD also led to increased IFN-γ secretion in both experimental CARs compared to CD19 CAR. To evaluate apoptosis in tumor cells following MV infection and CAR-T treatment, Annexin V near-infrared (NIR) was added to the culture medium, and the total NIR+ area was analyzed. MeVac-VLQSQRTD and MeVac-SGKALVLQSQRTD infection of U-87 MG resulted in significantly increased Annexin V staining when combined with 8-VHVL and 8-VLVH CAR-T cells (Figure 4F); however, CD19 CAR-T cells did not cause increased cytotoxicity. Kinetics in the early stages of CAR-T killing were slightly enhanced by MeVac-VLQSQRTD infection compared to MeVac-SGKALVLQSQRTD, likely resulting from higher affinity toward the shorter peptide. Additionally, control MeVac-eGFP did not result in differences regarding the killing of U-87 MG cells when treated with either CAR. These observations were in line with results using stably transduced U-87 MG (Figure S5A) and MeWo (Figure S5B). Similarly, upon infection of MeWo cells, MeVac-VLQSQRTD led to significantly increased killing when combined with 8-VHVL CAR-T, although not when combined with 8-VLVH CAR-T (Figure 4G). Strikingly, onset of killing after infection with MeVac-VLQSQRTD was faster than that for MeVac-SGKALVLQSQRTD, even though the global cytopathic effect elicited by MeVac-SGKALVLQSQRTD was more clearly visible.

### Addition of CAR-NK cells enables enhanced cytotoxicity mediated by CAR-T cells in MV-infected tumor cells

**Figure 5:**
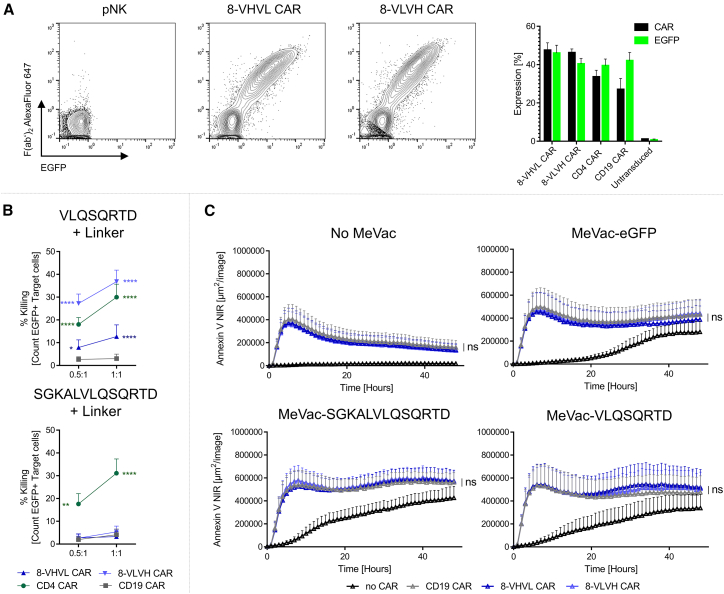
CAR-NK cell cytotoxicity is enhanced through inoculation of tumor cells with MV (A) Expression of CAR molecules on NK cells. Left panel shows representative plots of flow cytometry data of pNK, 8-VHVL CAR-NK, and 8-VLVH CAR-NK. Right panel shows expression of both experimental CARs (8-VHVL and 8-VLVH), CD4 and CD19 CAR on NK cells. Black bars represent CAR expression detected via anti-mouse F(abʹ)_2_ antibody, and green bars represent EGFP signal; data from *n* = 15 independent experiments are depicted as mean (SEM). (B) CAR-NK cells were cocultured with T cells from the same donor transduced to express VLQSQRTD or SGKALVLQSQRTD both with the rigid linker. T cells used as target cells were labeled with CellTrace Violet, and killing was assessed via PI staining. Only EGFP+ target T cells were analyzed for PI staining. Killing is depicted as percentage of PI+ cells in cocultures and independently cultured target T cells. Data from *n* ≥ 5 independent experiments are shown as mean (SEM). (C) U-87 MG cells were inoculated with MeVac-eGFP, MeVac-SGKALVLQSQRTD, or MeVac-VLQSQRTD. Forty-eight hours after inoculation, CAR-NK cells were added and apoptosis was monitored by addition of Annexin V NIR dye to the culture medium. Data from *n* = 4 independent experiments are shown as mean (SEM). Statistical analysis of (B) was performed by two-way ANOVA followed by Dunnett’s multiple-comparisons test, and experimental groups were compared to CD19 CAR. Statistical analysis of (C) was performed by two-way repeated measures ANOVA with Tukey’s multiple-comparisons test. ∗ = *p* ≤ 0.05, ∗∗ = *p* ≤ 0.01, ∗∗∗ = *p* ≤ 0.001, and ∗∗∗∗ = *p* ≤ 0.0001.

Beyond CAR-T cell therapies, NK cell-based treatments have gained increasing attention due to promising results in recent years.^33^^,^^34^ To study the potential benefits of NK cell therapy combined with oncolytic virotherapy, we first assessed the potency of CAR-NK cells targeting the peptide epitope VLQSQRTD. Therefore, we transduced primary NK cells resulting in CAR expression comparable to T cells (Figure 5A). We next conducted a cytotoxicity assay by coculturing CAR-NK cells with T cells from matched donors transduced to express VLQSQRTD-Linker, as described earlier. Among the CAR-NK groups, 8-VLVH CAR-NK exhibited the most potent killing activity, surpassing the cytotoxicity observed with CD4 CAR-NK cells (Figure 5B). In contrast, 8-VHVL CAR-NK cells showed lower killing efficacy. Notably, no cytotoxic activity was detected against target cells expressing the elongated SGKALVLQSQRTD-Linker, aligning with the findings from CAR-T cell assays conducted earlier (Figure 3D).

**Figure 6:**
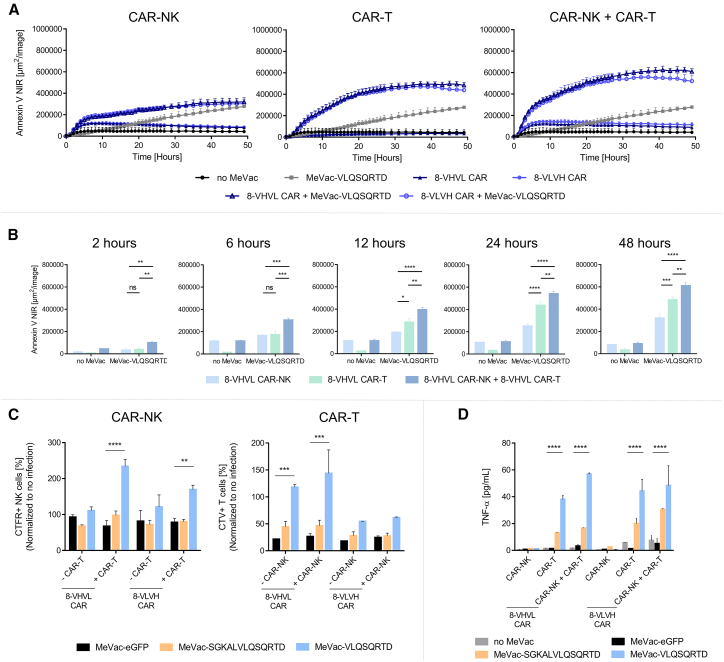
CAR-T cell cytotoxicity is enhanced via activation of CAR-NK cells by MV-infected tumor cells (A) Killing of U-87 MG cells inoculated with MeVac and then cocultured with 8-VHVL and 8-VLVH CAR-NK, CAR-T, or both CAR-NK and CAR-T cells is shown. Annexin V NIR dye was used to identify apoptotic cells. Data are shown as area per image and are depicted from *n* = 4 independent experiments as mean (SEM). (B) Annexin V NIR signals from plots in (A) are shown at five different time points over the course of the experiment. Values are shown as mean (SEM). (C) Detected cell counts of CellTrace FarRed+ (CTFR+) CAR-NK cells in coculture with U-87 MG after 96 h in absence or presence of CAR-T cells (left plot) and CellTrace Violet+ (CTV+) CAR-T cells in coculture with U-87 MG in absence or presence of CAR-NK cells (right plot) normalized to cell counts of samples without MeVac infection are shown as mean (SEM) from *n* = 2 independent experiments. (D) U-87 MG cells were inoculated with MeVac and then cocultured with 8-VHVL and 8-VLVH CAR-NK, CAR-T, or both CAR-NK and CAR-T cells. After 72 h, cell culture supernatant was harvested and TNF-α release was analyzed. Data are shown as mean (SEM) from *n* = 2 independent experiments. Statistical analysis of (A) was performed by two-way repeated measures ANOVA with Tukey’s multiple-comparisons test. Statistical analysis of (B), (C), and (D) was performed by two-way ANOVA with Tukey’s multiple-comparisons test. ∗ = *p* ≤ 0.05, ∗∗ = *p* ≤ 0.01, ∗∗∗ = *p* ≤ 0.001, and ∗∗∗∗ = *p* ≤ 0.0001.

To build on these findings, we infected U-87 MG and MeWo with MV and introduced CAR-NK cells 48 h post-inoculation. When combined with MV, CAR-NK cells were activated against virus-infected cells, resulting in prolonged and consistent cytotoxic responses against U-87 MG cells over time. In contrast, cytotoxicity swiftly declined without viral infection. Importantly, no CAR-specific effects were observed, as killing activity of NK cells was comparable across 8-VHVL CAR, 8-VLVH CAR, and CD19 CAR, regardless of the measles vector used (Figure 5C). MeWo displayed similar results, without significant CAR-dependent effects (Figure S6). Given the robust antiviral response observed in NK cells, we next evaluated their potential to enhance CAR-T cell activity. To test this, CAR-NK cells were premixed with CAR-T cells at a ratio of 1:10 and coapplied to U-87 MG cells infected with MeVac-VLQSQRTD. While the lower CAR-NK cell ratio (one-tenth of previous experiments shown in Figure 5C) resulted in reduced cytotoxicity in the CAR-NK only control condition, their cytotoxic activity against virus-infected tumor cells persisted over time (Figure 6A). CAR-T cells retained their cytotoxic effect as previously observed for both 8-VHVL and 8-VLVH CAR-T cells, despite half the effector cell count (Figure 4C). Notably, combining CAR-T and CAR-NK cells enhanced tumor cell killing. In MeVac-VLQSQRTD-infected U-87 MG, the addition of CAR-NK cells accelerated the onset of cytotoxicity compared to either cell type alone (Figure 6A). This effect was evident from the start of co-incubation, with combined CAR-T/-NK cell populations demonstrating superior cytotoxicity throughout the assay (Figure 6B). Early on, CAR-NK cells outperformed CAR-T cells due to their activation in response to virus-infected cells, but, from 12 h onward, CAR-T cells displayed greater killing efficacy, ultimately surpassing CAR-NK activity. Comparable results were obtained from experiments conducted with MeWo, albeit with less distinctive differences (Figure S7). Therefore, presence of CAR-NK cells amplified CAR-T cell responses, resulting in faster kinetics and sustained cytotoxicity.

The ratios of cell numbers in MV-treated against non-infected samples were analyzed after 96 h of coculture with U-87 MG. Here, both 8-VHVL and 8-VLVH CAR-NK cell numbers were increased in the presence of CAR-T cells in MeVac-VLQSQRTD-infected samples compared to CAR-NK only. Cell numbers were significantly higher than those for MeVac-eGFP- and MeVac-SGKALVLQSQRTD-infected samples. Similarly, detected numbers of CAR-T in the presence of CAR-NK cells were elevated in MeVac-VLQSQRTD-infected samples (Figure 6C). Generally, 8-VHVL CAR-NK and CAR-T cells showed more pronounced increases than 8-VLVH CAR-NK and CAR-T cells. Increased ratios of cell numbers were also detected in cultures with MeWo, although the effect of coculturing CAR-NK and CAR-T was not as prominent as that in U-87 MG (Figure S8). We next quantified various cytokines and chemokines released into the cell culture supernatant to study further potential interactions between CAR-NK and CAR-T cells. Here, we observed increased levels of tumor necrosis factor alpha (TNF-α) in MeVac-VLQSQRTD and to some extent in MeVac-SGKALVLQSQRTD-infected U-87 MG. Cultures containing both CAR-NK and CAR-T cells showed higher levels of TNF-α compared to CAR-T alone, while CAR-NK did not show TNF-α release (Figure 6D). In addition, interleukin (IL)-2, granulocyte-monocyte colony stimulating factor (GM-CSF), granzyme B, IFN-γ, and IL-4 were specifically released in response to infection with MeVac-VLQSQRTD and MeVac-SGKALVLQSQRTD (Figure S9). Perforin showed slightly increased levels when CAR-NK and CAR-T cells were applied together compared to culture with either immune cell type alone. IL-6 and monocyte chemoattractant protein 1 (MCP-1) did not show immune effector cell-specific patterns while IL-10, IL-17A, and IL-21 were not detected (data not shown). To mechanistically study the interaction of CAR-NK and CAR-T cells, we investigated numerous activating receptors and ligands shaping the interactions between T and NK cells by flow cytometry. Interestingly, we observed elevated levels of OX40 on CAR-T cells for both cocultures with U-87 MG and MeWo infected with MeVac-VLQSQRTD and MeVac-SGKALVLQSQRTD. OX40L was in turn expressed on CAR-NK cells and induced during coculture with MeWo cells in the presence of CAR-T cells (Figure S10). Apart from OX40, 2B4 and partially NKG2D showed increased surface expression on CAR-T cells in response to MeVac-VLQSQRTD- and MeVac-SGKALVLQSQRTD-infected targets. These results highlight the complementary strengths of CAR-T and CAR-NK cells, with their combined use providing a potent and sustained antitumor effect.

## Discussion

Based on our hypothesis that shedding of stress-induced ligands on tumor cells generates peptide remnants, which may function as targetable neoepitopes, we generated antibodies against an MICB-derived octapeptide with prevalence in malignant and stressed cells. These antibodies were subsequently used for the formation of two distinct CAR molecules. Targeting cells either transduced to stably express an octapeptide-EGFP fusion protein or inoculated with MV encoding the same protein, we have shown specific antigen-directed cytotoxicity elicited by CAR-T cells upon membrane-distal positioning of the octapeptide through the insertion of a rigid linker. The search for such neoepitopes, which can be safely targeted in solid tumors using CAR therapies, is challenging. To address this, we took advantage of proteolytic processes regularly occurring in the TME. Within the innate immune system, part of immunosurveillance is the recognition of MICA/B by NKG2D as an activating receptor on NK and a co-activating receptor on T cells. However, through shedding of MICA/B, cancer cells can escape this mechanism of immunosurveillance.^7^ So far, the successful generation and effect of therapeutic antibodies inhibiting the shedding of MICA/B, thereby promoting NK cell-driven tumor immunity, has been described.^9^ In the present study, we focused on the shedding process as a starting point by targeting the peptide remaining on the cellular surface as a neoepitope. MICA/B proteolytic cleavage is a complex process involving multiple steps and potential cleavage sites. First, a disulfide bond within the alpha-3 domain between amino acid residues 202 and 259 has to be removed.^35^ Then, MMPs can cleave MICA/B either in the alpha-3 domain or in the linear stalk between the alpha-3 and the transmembrane domain. Waldhauer et al. previously identified several C termini of shed MICA/B through mass spectroscopy located within the alpha-3 domain.^6^ Consequentially, in this study, we designed an octapeptide starting at position V302, mimicking the membrane-bound stalk after enzymatic cleavage in the alpha-3 domain. Using hybridoma technique, we immunized mice with the octapeptide VLQSQRTD and successfully generated two antibody clones, 8E1 and 12F7. During ELISA screening, we included full-length MICB to exclude antibodies binding the native protein at the polyclonal stage, thereby minimizing the risk of the final product targeting non-malignant cells.

Epitope mapping identified the highly specific 4-mer sequence QSQR required for high-affinity binding of both antibody clones 8E1 and 12F7. Upstream of the N-terminal valine in the VLQSQRTD octapeptide used in our study, no clear preference for specific amino acids was observed, while the N-terminal valine represented a conserved residue in around 50% of all sequences. This suggests that both antibodies can potentially bind various MICB-derived peptides generated through shedding, thereby expanding their therapeutic range. To further investigate this, we incorporated an N-terminally elongated version of the octapeptide starting from S297 as a second artificial product of shedding in this study. Elongating the peptide reduced affinity, as indicated by a K_D_ value of 214.8 μM measured for the SGKALVLQSQRTD peptide versus 3.85 nM for the VLQSQRTD peptide for clone 12F7. Consequently, peptides of intermediate lengths between those used in this study are expected to exhibit K_D_ values with lower affinity depending on the accessibility of amino acids necessary for binding.

Upon translation of these findings, we observed low MFI on A375 cells stably expressing the VLQSQRTD octapeptide fused to the TM and intracellular domains of MICB and an EGFP, suggesting low antigen affinity. Here, binding of clone 8E1 was considerably lower than that of clone 12F7. On the cell surface, the peptide is located in a complex environment surrounded by other proteins, glycoproteins, and lipids. This crowded molecular landscape can create physical barriers that hinder antibody access to its target epitope as opposed to high accessibility in solution.^36^ Conformational changes of the peptide likely do not play a role in this study, as the octapeptide is not expected to form structures masking the availability of the binding site. On the other hand, in cells transduced to stably overexpress the antigen, antigen density on the cell surface can negatively affect antibody binding as closely spaced epitopes may prevent simultaneous occupancy of all binding sites due to steric hindrance.^37^ A study from Phung et al. describes the difficulty of isolating high-affinity monoclonal antibodies directed against tumor antigens on the cell surface, where binding efficiency often diminishes compared to purified antigens.^38^ This issue becomes particularly critical in our study, as the target antigen is located proximal to the membrane, further lowering accessibility.

Due to these findings, we adapted our approach of how to apply the octapeptide VLQSQRTD as a neoepitope. By introducing a rigid (EAAAK)_4_A linker between the peptide sequence derived from the extracellular domain of MICB and the transmembrane domain, we demonstrated increased antigen availability and improved detection by both clones on Mac-1 transduced to stably express the neoepitopes. We then used the sequences of heavy and light chains of clone 12F7 to develop CARs directed against the VLQSQRTD peptide. We synthesized two CARs with alternating orientation of heavy and light chains, resulting in CAR molecules 8-VHVL and 8-VLVH. The positioning of CDRs, influenced by heavy- and light-chain orientation, can affect the specificity and efficiency of antigen recognition.^39^ This is particularly relevant because antigen affinity is a critical parameter for CAR function, determining the ability to recognize antigens, induce CAR signaling, and activate effector cells. Importantly, the affinity must be high enough to activate CAR-T cells but not so high that it triggers activation-induced cell death or toxicity.^3^ Previous studies, such as those by Ochi et al., have shown that, independently of the V_H_ and V_L_ chain order, CARs targeting the NY-ESO-1 antigen delivered robust function.^40^ We also observed specific CAR-T cell activation and selective killing upon encountering the antigen with both 8-VHVL and 8-VLVH CAR. However, the orientation of heavy and light chains within the scFv impacted the CAR’s performance. Specifically, 8-VLVH CAR-T exhibited enhanced killing of transduced Mac-1 stably expressing either the VLQSQRTD-Linker or the SGKALVLQSQRTD-Linker. On the other hand, reduced expression of the target antigen in non-puromycin-selected T cells still caused efficient killing in VLQSQRTD-Linker transduced cells, while cytotoxicity in T cells expressing the SGKALVLQSQRTD-Linker was almost absent. Additionally, increased CD137 and LAG-3 were observed upon cocultivation with T cells, independently of the transgene. The application of 8-VHVL CAR did lead to reduced cytotoxicity in both Mac-1 and T cells compared to 8-VLVH CAR, staying consistent also after 72 h of coculture. No killing of SGKALVLQSQRTD-Linker transduced T cells could be observed. Intracellular signaling of CAR-T cells was particularly sensitive to the chain order within the CAR. In this study, 8-VLVH CAR-T consistently demonstrated higher CD137 expression and IFN-γ release in response to VLQSQRTD-Linker, aligning with its superior cytotoxicity. In contrast, 8-VHVL CAR-T, likely due to lower antigen affinity, showed reduced intracellular signaling despite achieving effective target cell killing. Similar findings have been reported by other studies, where CAR affinity influenced cytokine production and T cell activation.^41^^,^^42^ These results demonstrate that both chain order and antigen affinity significantly impact the cytotoxic potential and signaling efficacy of CAR-T cells.

The inclusion of a rigid linker between the extracellular and transmembrane regions of MICB creates a structure not found physiologically and enhances the potential of the octapeptide as a targetable neoepitope. This approach addresses the lack of suitable natural targets for CAR therapy in solid tumors. Here, OVs emerge as promising tools due to their multifunctionality. They preferentially infect tumor cells while enabling the expression of therapeutic transgenes.^27^^,^^28^ Several preclinical studies incorporating adenovirus, vaccinia virus, or herpes virus harness the viruses’ vector capabilities, often in combination with CAR-T cells targeting antigens like BCMA or CD70.^43^ One ongoing clinical study, for instance, investigates HER2 CAR-T cells combined with oncolytic and helper-dependent adenovirus encoding IL-12 and anti-PD-L1 for the treatment of solid tumors (NCT03740256). In our experiments, we tested multiple MOIs of a measles vector encoding GFP on highly susceptible U-87 MG and moderately susceptible MeWo, demonstrating potent cytopathic effects. Using a measles vector encoding the membrane-anchored octapeptide VLQSQRTD-Linker—MeVac-VLQSQRTD—we determined that an MOI of 1 was optimal for achieving detectable expression of VLQSQRTD after 48 h while avoiding excessive cytopathic effects. Infection with MV and subsequent type I IFN signaling concomitantly induce upregulation of checkpoint receptor ligands such as PD-L1. Therefore, combinations of oncolytic MV and PD-1/PD-L1 checkpoint blockade can yield superior therapeutic outcomes.^44^^,^^45^

In pre-experiments using U-87 MG and MeWo cells stably expressing GFP, VLQSQRTD-Linker, or SGKALVLQSQRTD-Linker, we observed efficient killing of tumor cells by both 8-VHVL and 8-VLVH CAR-T upon encountering either peptide (Figure S4). Based on these findings, we hypothesized that infection with MV leading to transgenic expression of the neoepitope will result in efficient killing of infected but not yet lysed tumor cells. Our experiments confirmed that tumor cells did not exhibit cytopathic effects 48 h post-inoculation. Additionally, infected tumor cells exhibited significantly higher cell death when infected with both MeVac-VLQSQRTD and MeVac-SGKALVLQSQRTD compared to MeVac-eGFP when cocultured with 8-VHVL or 8-VLVH CAR-T cells, relative to CD19 CAR-T cells. Clinical data indicate that MV may facilitate T cell priming against tumor antigens.^46^ Notably, U-87 MG cells were more efficiently killed after infection with MVs encoding either neoepitope through both experimental CARs, while MeWo cells were only killed more efficiently upon infection with MeVac-VLQSQRTD, not MeVac-SGKALVLQSQRTD, compared to CD19 CAR-T cells. This difference can be partially explained by a slightly higher cytopathic effect induced by MeVac-SGKALVLQSQRTD in this batch of experiments, despite using equivalent MOIs. Additionally, MeWo cells exhibited lower neoepitope expression, resulting in reduced antigen availability. A notable observation was the reduced killing efficiency of 8-VLVH CAR-T compared to 8-VHVL CAR-T on MeWo cells post-infection. This could be attributed to higher background killing by CD19 CAR-T cells in these assays, which limited the relative increase in killing by 8-VLVH CAR-T cells. Interestingly, apoptosis levels in U-87 MG cells did not differ between infections with MeVac-VLQSQRTD and MeVac-SGKALVLQSQRTD and subsequent incubation with 8-VHVL and 8-VLVH CAR-T cells. However, IFN-γ levels were increased between 2- to 4-fold following inoculation with MeVac-VLQSQRTD. This disparity between cytotoxic effects and the intrinsic signaling of CAR-T cells suggests that even minimal CAR engagement is sufficient to effectively kill infected target cells expressing the SGKALVLQSQRTD peptide; however, it does not support the robust downstream signaling required for release of high levels of IFN-γ.^41^^,^^42^

In addition to tumor cell infection, OVs elicit immunogenic cell death and local inflammation, accompanied by the release of DAMPs and pathogen-associated molecular patterns (PAMPs) from lysed cells, in turn leading to the recruitment of cells of the innate immune system to the TME.^15^ In consequence, NK cells are able to eliminate infected or malignant cells and could contribute to antitumor immunity in oncolytic virotherapy. The critical role of NK cells in tumor control is underscored by observations that tumors are often more aggressive in the absence of functional NK cells. Conversely, studies have shown that NK cell infiltration and functionality in solid tumors are positively correlated with improved prognosis.^47^ Based on these considerations, we evaluated the efficacy of CAR-NK cells against the neoepitope. CAR-NK cells demonstrated potent and specific killing of donor-matched T cells transduced to express VLQSQRTD-Linker. Interestingly, no killing was detected in target cells expressing SGKALVLQSQRTD-Linker, likely due to the CARs’ reduced affinity for the peptide and lack of NK-specific signaling optimization. This limitation may potentially be overcome by tailoring CAR design to better suit NK cell signaling requirements.^48^ When we incubated tumor cells with MV and CAR-NK cells, we did not observe any CAR-dependent benefit, likely overshadowed by the strong natural antiviral response. NK cells showed prolonged cytotoxic activity in response to viral infection, implying that OVs can overcome NK cell exclusion and hyporesponsiveness in the TME. This likely not only enhances NK cell recruitment to the tumor site but also sustains their activation, improving antitumor responses. By modulating the TME, virotherapy holds the potential to restore NK cell functionality and improve outcomes in solid malignancies.^49^^,^^50^

To investigate potential synergy between immune cells, we conducted coculture experiments with CAR-T and CAR-NK cells. CAR-NK cells enhanced CAR-T cell responses, sustaining cytotoxicity and priming them for earlier and stronger activation. Time-point analysis revealed that CAR-NK cells initiated rapid killing in both mono- and cocultures, driven by innate receptors that distinguish infected or malignant from healthy cells.^51^ Although CAR-NK cells lacked CAR-dependent killing, their innate activity contributed to more durable CAR-T cell-mediated cytotoxicity. This synergy is consistent with the superior efficacy of combined CAR-NK and CAR-T cell treatments.^52^ NK cells enhance T cell activation, tumor-directed migration, and fitness by reducing exhaustion and senescence. In tumor models and translational studies, especially with OV priming, NK/T combination therapies boost effector expansion, persistence, and function, which is reflected in increased cell numbers detected upon MeVac-VLQSQRTD exposure.^53^^,^^54^^,^^55^ One possible pathway of NK/T interaction is via OX40, an inducible costimulatory receptor on activated T cells, while OX40L on activated NK cells serves as its cognate ligand. Engagement provides stimulation to T cells, while OX40L engagement can reciprocally activate NK cells via reverse signaling.^56^ The OX40-OX40L axis is known to enhance cytokine production, including TNF-α and IFN-γ, providing a mechanistic link between phenotypic and functional observations and reflecting synergistic activation of CAR-NK and CAR-T cells.^57^^,^^58^ NK cells thus serve as effective bystanders within the TME, complementing CAR-T cell activation and proliferation, which often lacks efficacy when induced by OVs alone.^49^^,^^54^ To deepen the understanding of mechanistic interactions between NK cells and CAR-T cells in the presence of OV, it will be crucial to perform additional assays, particularly those incorporating spheroid tumor models and *in vivo* systems that simulate the presence of bystander NK cells.

In conclusion, the results of this study highlight the complementary strengths of CAR-T and (CAR-)NK cells, especially in combination with oncolytic virotherapy, leading to a potent and sustained antitumor effect. Additionally, this study shows that the expression of a membrane-anchored octapeptide neoepitope mediated by measles vaccine vectors is a viable starting point for developing more effective combination therapies, possibly overcoming current challenges in the treatment of solid tumors.

## Materials and methods

### Development of antibodies against an MICB-derived peptide

Antibodies directed against an octapeptide stalk of MICB were generated using the hybridoma technology and was carried out by ProteoGenix SAS (Schiltigheim, France). Five mice were immunized with KLH-conjugated VLQSQRTD-C peptide using complete Freund’s adjuvant for the initial immunization and incomplete Freund's adjuvant (IFA) adjuvant for all further immunizations. A total of four immunizations were performed over a time period of 42 days. Positive screening was performed via ELISA using VLQSQRTD-C and VLQSQRTD-KKC peptides while negative screening was done using an MICB-Fc fusion protein which was purified via Protein G PLUS-agarose (Santa Cruz Biotechnology, Dallas, TX, USA).

Nine days after the final boost, fusions of splenocytes from two mice with a myeloma cell line were performed to create hybridomas. Hybrid cells underwent HAT selection, and monoclones were expanded and screened via ELISA. Briefly, eleven clones reacting to the immunization antigen were identified by ELISA. Screening of polyclonal cell culture supernatant on A375 cells stably expressing the octapeptide fused to the TM and intracellular domains of MICB and an EGFP was used as an additional verification step. In short, A375 cells overexpressing the VLQSQRTD octapeptide, the N-terminally elongated SGKALVLQSQRTD peptide, or full-length MICB were incubated with 100 μL of hybridoma culture supernatant on ice for 60 min. After three washing steps using PBS with 5% fetal calf serum (FCS) and 0.1 mM EDTA, a secondary staining with Alexa Fluor 647-conjugated AffiniPure F(ab’)_2_ fragment goat anti-mouse immunoglobulin G (IgG) and F(ab’)_2_ fragment-specific antibody (Jackson ImmunoResearch, West Grove, PA, USA) for 30 min at 4°C, followed by two washing steps, was carried out. In consequence, four clones were determined to undergo further sub-cloning. Two rounds of sub-cloning were performed for clone #14 while four rounds of sub-cloning were performed for clones #8, #9, and #12. Finally, after re-evaluating monoclonal cell culture supernatants and performing positive and negative screening via ELISA, clones 8-E1-1-C1-D1 and 12-F7-C12-E3 were chosen for purification by protein A/G. Two mg of antibody were retrieved and shipped to our institute for further analysis.

### Surface Plasmon Resonance

Antibody kinetics to the VLQSQRTD octapeptide and the N-terminally elongated peptide SGKALVLQSQRTD were analyzed using SPR adapted from Köppen et al.^59^ Briefly, using a Sierra SPR-24 Pro (Bruker, Ettlingen, Germany), both antibodies 8-E1-1-C1-D1 and 12-F7-C12-E3 were used at dilutions of 2 μg/mL in HBS-EP buffer (GE Healthcare, Düsseldorf, Germany) at a pH of 7.4 and immobilized onto an IgG-capture-sensor-chip (Bruker, Ettlingen, Germany) at a flow rate of 30 μL/min using HBS-EP as a running buffer. For both antibodies, a single-injection-cycle kinetic analysis was carried out by applying 0.5–100 nM of the octapeptide and 1–200 μM of the 13-aa peptide. SPR sensorgrams were evaluated using the single-cycle kinetic model.

### Peptide-phage display

To investigate antibody binding patterns to the octapeptide antigen, a statistical phage display approach was applied on purified antibody samples as described previously by Kern et al.^60^ Briefly, a trinucleotide-based, 16 mer peptide-phage display library covering more than 10^9^ clones was used for two rounds of panning against each individual antibody. Datasets from each sequencing run were cleared from sequencing errors when read into the latest version of LibDB software (Epitopic GmbH, Leipzig, Germany). First, statistics of all motifs in the datasets were calculated, comparing their occurrence to the amino acid distribution expected from the library design. Since the library has a predictable statistical distribution, any enrichment likely results from the selection experiment. Sequences with motifs that resemble the antigen were significantly enriched in the sequence pool. Their alignment with the antigen sequence revealed relevant positions and amino acid preferences for the antibodies. WebLogo 3.7.12 was used for visualizing consensus peptide sequences.

### Cell culture

#### Primary cells

Buffy coats from healthy donors were obtained from the Institute for Transfusion Medicine of Leipzig University Hospital in Leipzig, Germany (ethical vote number 327/22-ek). Cells were then isolated by standard density-gradient centrifugation using Ficoll-Paque (VWR, Darmstadt, Germany, catalog no. 17-1440-03) using the RosetteSep human NK cell enrichment cocktail (STEMCELL Technologies, Vancouver, Canada) for NK cells and the RosetteSep human T cell enrichment cocktail for T cells. NK cells were cultured at 1 × 10^6^ cells/mL in NK MACS medium (Miltenyi Biotec, Bergisch Gladbach, Germany) with 5% human AB serum (Sigma-Aldrich, St. Louis, MO, USA), 500 U/mL IL-2, and 140 U/mL IL-15 (PeproTech, New Jersey, NJ, USA). T cells were cultured at 1 × 10^6^ cells/mL in T cell medium (Thermo Fisher Scientific, Waltham, MA, USA) with TransAct (Miltenyi Biotech, Bergisch Gladbach, Germany) and 500 U/mL IL-2. TransAct was removed after 24 to 72 h, and cells were cultured using only 500 U/mL IL-2 at 1 × 10^6^ cells/mL.

#### Cell lines

Primary, CD4+ cutaneous T cell lymphoma Mac-1 cells (DSMZ, Braunschweig, Germany) were cultured in RPMI + 10% (v/v) FCS (Bio&SELL, Feucht, Germany) and 1% (v/v) penicillin-streptomycin (Thermo Fisher Scientific, Waltham, MA, USA). A375 cells, U-87 MG glioblastoma (Sigma-Aldrich, St. Louis, MA, USA), and MeWo melanoma (CLS, MEOS Erfurt, Germany) were cultured in DMEM with 10% FCS and 1% penicillin-streptomycin.

### Construction of Lentiviral Vectors

The coding sequence of human MICB was amplified from PBMC cDNA using the following primers: MICB_fw (5′-ATATCTAGAGCCGCCACCATGGGGCTGGGCCGGGTC-3′) MICB_rev (5′-TAGACgaattcCTAGGTGCCCTCAGTGGAACC-3′). The gene was cloned into the lentiviral expression vector hEF1α-H2B-mVenus-IRES-mChery-PGK-Puromycin (Addgene #99278, kindly provided by Jordan Miller) for initial sequencing.^61^

All other lentiviral constructs were cloned into a pTwist-CMV-BetaGlobin-WPRE-Neo backbone (Twist Bioscience, USA). Human MICB N-terminally (extracellularly) coupled to TagRFP and C-terminally (intracellularly) coupled to EGFP was originally ordered from Twist Bioscience (San Francisco, CA, USA). This MICB sequence was used as a basis for cloning the VLQSQRTD and SGKALVLQSQRTD peptides into the same pTwist backbone.

In addition, two constructs linking the coding sequence of both peptides to the transmembrane and intracellular domain of human MICB via an (EAAAK)4A linker were created.^62^ The 5′ primers were designed including the sequence of the native MICB signal peptide, and the 3′ primer binds to the 3′ end of the intracellular EGFP.

Briefly, PCR products were purified using a QIAGEN gel DNA recovery kit according to the manufacturer’s instructions (QIAGEN, Germantown, MD, USA). Ligations were performed with 50 ng vector and a molar insert to vector ratio of 3:1 using T4 DNA ligase overnight at 16°C according to the manufacturer’s instructions (NEB, Ipswich, MA, USA). Five μL of the ligation mixture was then added to 20 μL of chemical competent *E. coli* TOP10 (Thermo Fisher Scientific, Waltham, MA, USA) for transformation and incubated on ice for 30 min followed by a heat shock of 30 s at 42°C and 2 min on ice. Then, 150 μL of Super Optimal broth with Catabolite repression (SOC) medium was added, and *E. coli* were incubated at 37°C for 1 h while shaking. Afterward, 100 μL was plated on LB agar plates containing 50 μg/mL ampicillin. Colonies were grown overnight at 37°C. Colonies were picked and transferred to 5 mL LB medium containing 50 μg/mL ampicillin. The next day, plasmids were isolated using the ZymoPURE miniprep kit according to the manufacturer’s instructions (Zymo Research, Irvine, CA, USA).

The plasmid pCDH-CMV-MCS-EF1-GFP+Puro was used as a control (System Biosciences, Palo Alto, CA, USA).

### Construction of gammaretroviral vectors

All retroviral CAR constructs are second-generation constructs containing the respective scFv targeting CD4 (scFv derived from the humanized MAX16.H5 antibody),^63^ CD19 (scFv derived from the clone FMC63), and the VLQSQRTD octapeptide in two configurations VHVL (a-8-VHVL) and VLVH (a-8-VLVH). Variable heavy (5′-CAGGTCCAGCTGCAGCAGTCTGGAGCTGAGCTGGTCAGGCCTGGGACTTCAGTGAAGGTGTCCTGCAAGGCTTCTGGATACGCCTTCACTAATTACTTGATAGAGTGGATAAAGCAGAGGCCTGGACAGGGCCTTGAGTGGATTGGAGTGATTAATCCTAGAAGTGGTGGTACTAACTACAATGAGAAGTTCAAGGGCAAGGCAACACTGACTGCAGACAAATCCTCCATCACTGTCTACATGCAGCTCAGCAGCCTGACATCTGATGACTCTGCGATCTATTTCTGTGTAAGATCGGAGAGTTACGGTGATTACGGAGCCTGGTTTGCTTACTGGGGCCAAGGGACTCTGGTCACTGTCTCTGCA-3′) and light (5′-GACATTGTGCTGACCCAATCTCCAGCTTCTTTGGCTGTGTCTCTAGGGCAGAGGGCCACCATATCCTGCAGAGCCAGTGAAAGTGTTGATAGTTATGGCAATAGTTTTATGCACTGGTACCAGCAGAAACCAGGACAGCCACCCAAACTCCTCATCGATCGTGCATCCAACCGAGAATCTGGAATCCCTGCCAGGTTCAGTGGCAGTGGGTCTAGGACAGACTTCACCCTCACCATTAATCCTGTGGAGGCTGATGATGTTGCAACCTATTACTGTCAGCAAAGTAATGAGGACCCATTCACGTTCGGCTCGGGGACAAAGTTGGAAATAAAG-3′) chains were connected using a (G4S)3 linker sequence. Downstream of the scFv domain, the IgG1 hinge region was inserted, followed by a CD8a transmembrane domain, a CD28 costimulatory domain, and a CD3ζ endodomain. GFP was connected using a 2A protein (P2A) cleavage site. These constructs were cloned into the gammaretroviral vector pBullet (kindly provide by Reno Debets, Erasmus University Medical Center).^64^

### Plasmids and virus production

#### LVs

LV-containing supernatants were generated via transient transfection of HEK293T cells with the use of TransIT transfection reagent (Mirus Bio, Madison, WI, USA) according to the manufacturer’s instructions. Briefly, 1.5 × 10^5^ HEK293T cells were seeded per well in a 6-well plate 1 day prior to transfection. For LV production, on the day of transfection, 1 μg of the packaging plasmids in a 4:1:1 mixture (pMDLg/pRRE-gagpol:pRSV-Rev:Env—i.e., 0.667 μg pMDLg/pRRE-gagpol and 0.167 μg of pRSV-Rev and baboon endogenous retrovirus envelope [BaEV] each) and 1 μg of the transgene plasmid were mixed in 200 μL DMEM (Thermo Fisher Scientific, Waltham, MA, USA), and 6 μL of TransIT transfection reagent was added for production of LVs. Third-generation packaging plasmids pMDLg/pRRE-gagpol and pRSV-Rev (Addgene #12251 and #12253, kindly provided by Didier Trono) were used alongside the transfer vector. For transduction of cell lines, VSV-G pseudotyped LV (pCMV-VSV-G was a gift from Bob Weinberg, Addgene plasmid #8454) was used. The LV-containing cell culture supernatant was collected 48 h after transfection and filtered using a 0.45 μm syringe filter. Supernatants were either directly used for transduction of primary cells or stored at −80°C.

#### Gammaretroviral vectors

Gammaretroviral particles were generated via transient transfection of HEK293T cells with the use of TransIT transfection reagent. Briefly, 1.5 × 10^5^ HEK293T cells were seeded per well in a 6-well plate 1 day prior to transfection. On the day of transfection, retroviral transfer plasmids encoding CAR sequences were co-transfected with the viral packaging plasmids pHit60 and BaEV, and transgene plasmids were added in a 1:0.5:0.5 μg mixture to 200 μL DMEM with 6 μL of TransIT transfection reagent. Supernatant containing gammaretroviral particles was collected 48 h after transfection and filtered using a 0.45 μm syringe filter. Supernatant was either directly used for transduction of primary cells or stored at −80°C.

### Generation of CAR-T and CAR-NK cells

Primary human T cells and NK cells were transduced between day 3 and day 7 after isolation. 2.5 × 10^5^ cells were seeded per well in a 24-well plate. Five hundred μL of supernatant containing viral vectors was mixed with the transduction enhancer Vectofusin-1 (Miltenyi Biotec, Bergisch Gladbach, Germany) to a final concentration of 10 μg/mL. After spinfection at 37°C and 1,200×*g* for 90 min, 500 μL of the respective fresh complete culture medium was added to the transduced cells, and they were cultured in an incubator under standard conditions. Transduction efficiency was assessed via GFP reporter expression and by CAR detection through Alexa Fluor 647-conjugated AffiniPure F(ab’)2 fragment goat nti-mouse IgG, F(ab’)2 fragment-specific antibody (Jackson ImmunoResearch, West Grove, PA, USA) 3 to 4 days after transduction using flow cytometry (MACSQuant 10, Miltenyi Biotec, Bergisch Gladbach, Germany).

### Generation of target tumor cells

Mac-1, U-87 MG, MeWo, and A375 cells were transduced using VSV-G pseudotyped LV. To this end, 5 × 10^4^ cells were seeded per well in a 24-well plate. Five hundred μL of supernatant containing viral vectors was mixed with the transduction enhancer Vectofusin-1 to a final concentration of 10 μg/mL. After spinfection at 37°C and 400×*g* for 60 min, 500 μL of fresh complete culture medium was added to the transduced cells, and they were cultured in an incubator under standard conditions. Four days after transduction, tumor cells were subjected to puromycin selection for 14 days before further use.

For primary T cells, 2.5 × 10^5^ cells were seeded per well in a 24-well plate. Five hundred μL of supernatant containing viral vectors was mixed with the transduction enhancer Vectofusin-1 to a final concentration of 10 μg/mL. After spinfection at 37°C and 1,200×*g* for 90 min, 500 μL of fresh complete culture medium was added to the transduced cells, and they were cultured in an incubator under standard conditions.

### Recombinant MV

Oncolytic Schwarz strain measles vaccine (MeVac) vectors encoding the octapeptide and an N-terminally elongated peptide fused to MICB and EGFP, termed pcMeVac P-VLQSQRTD and pcMeVac P-SGKALVLQSQRTD, were designed as described by Heidbuechel and Engeland.^65^ Transgene inserts were amplified from plasmids VLQ-Stalk-EAAAK EGFP pTwist Lenti SSFV WPRE and SGK Long Stalk EAAAK EGFP pTwist Lenti SSFV WPRE, respectively, using primers 5‘ GCACGCGTGCCGCCACCATGGGGCTGG 3‘ (forward) and 5‘ CGGCGCGCTTACTTGTACAGCTCGTCCATGCCG 3‘ (reverse for VLQSQRTD) or 5‘ CGGCGCGCGGTTTACTTGTACAGCTCGTCCATGC 3‘ (reverse for SGKALVLQSQRTD) using Q5 Polymerase (NEB, Ipswich, MA, USA) according to the manufacturer’s instructions. After gel extraction, PCR products were cloned into pJET1.2/blunt using the CloneJET PCR Cloning Kit (Thermo Fisher, Waltham, MA, USA). After verification by Sanger Sequencing (Eurofins Genomics, Ebersberg, Germany), transgene inserts were excised from pJET1.2/blunt using MluI and BssHII (NEB, Ipswich, MA, USA). pcMeVac P-ATU, a MeVac antigenomic vector harboring an additional transcription unit downstream of the P open reading frame,^32^ was linearized with MauBI (Thermo Fisher, Waltham, MA, USA). Subsequently, the transgene inserts were ligated into linearized pcMeVac P-ATU. Dephosphorylation and ligation were carried out using Rapid DNA Dephos & Ligation Kit (Roche, Basel, Switzerland). Correct insertion of the transgenes was verified by Sanger sequencing.

For rescue of recombinant MeVac, following a protocol based on Mühlebach et al.,^66^ 8 × 10^5^ HEK293T cells/well were seeded in 2 mL DMEM + 10% FCS into 6-well plates. The next day, cells were transfected with MeVac antigenomic plasmids (4 μg) as well as plasmids encoding the MeVac ribonucleoprotein components, pcDiMER-N (0.4 μg), -P (0.1 μg), and –L (0.4 μg) as well as 0.1 μg pcDi dsRed (transfection control) using 12.5 μL Lipofectamine 2000 (Thermo Fisher Scientific, Waltham, MA, USA) in 200 μL DMEM. Prior to transfection, cells were washed with PBS and medium was changed to DMEM without additives. Four to 6 hours post-transfection, medium was changed to DMEM + 2% FCS. Two days after transfection, transfected HEK293T cells were completely suspended in their medium by pipetting up and down. The suspension was transferred onto Vero cells (0.5 × 10^6^ cells seeded one day prior to transfer in DMEM + 10% FCS into a 10 cm dish). After 3 to 10 days of incubation, when syncytia formation was observed, cells were harvested by scraping with a cell lifter, vortexed, and snap-frozen in liquid nitrogen. If cells were incubated for more than 4 days, cells were harvested by scraping. One-fourth of the cell suspension was transferred onto fresh Vero cells (0.5 × 10^6^ cells seeded one day prior to transfer in DMEM + 10% FCS into a 10 cm dish). The remaining cell suspension was snap-frozen in liquid nitrogen for 5 min, thawed in a 37°C water bath, and centrifuged at 5,000×*g* for 5 min at 4°C, and the supernatant was transferred to the 10 cm dish for incubation overnight.

For reference, a MV vector carrying a GFP transgene, MeVac-eGFP, first described by Veinalde et al., was employed.^32^ Viral titers were determined and recombinant MeVac was further propagated as described by Heidbuechel and Engeland.^65^ Recombinant viruses from passage 2 were used for experiments. The experimental MOI of each MeVac construct was determined by normalizing the MOI to the GFP expression measured via flow cytometry in infected cells 48 h after inoculation. To generate growth curves, cells were infected at MOI 3 and viral progeny were determined by titration as described previously.^65^

### Determination of CAR-T and CAR-NK cell functionality in coculture with suspension cells

Mac-1 cells or primary T cells were labeled with 1 μM of CellTrace Violet (Thermo Fisher Scientific, Waltham, MA, USA) according to manufacturer’s instructions. Briefly, staining was carried out for 20 min at 37°C, followed by addition of five times the volume of RPMI 1640 medium without phenol red (Thermo Fisher Scientific, Waltham, MA, USA) with supplementation of 10% FCS to stop the reaction. After labeling, Mac-1 or transduced primary T cells were cocultured with CAR-T or CAR-NK cells at effector:target (E:T) ratios of 0.5:1 and 1:1 for 16 h at 37°C and 5% CO2. After cocultivation, analysis was carried out using flow cytometry on a MACSQuant10 (Miltenyi Biotec, Germany) with PI solution (Miltenyi Biotec, Bergisch Gladbach, Germany) applied directly before each measurement to discriminate dead cells. The percentage of viable Mac-1 cells was assessed on the CellTrace+ PI-negative fraction, while viability of primary T cells was assessed as the absolute number on the EGFP+ PI-negative fraction within the CellTrace+ cells. The formula used to calculate cytotoxicity was

Killing(%)=100%−(PI−negativetargetsinsample/PI−negativetargetsincontrol)×100.

For evaluation of CAR-T cell activation, staining with CD137 (CD137 Antibody, PE-Vio770, anti-human, REAfinity) (Miltenyi Biotech, Bergisch Gladbach, Germany) was carried out after 24 h of co-incubation. For evaluation of CAR-T cell exhaustion, the monoclonal antibodies LAG-3 (CD223 Antibody, PE-Vio770, anti-human, REAfinity), PD-1 (CD279 Antibody, APC, anti-human, REAfinity), and TIM-3 (CD366 Antibody, PE-Vio615, anti-human, REAfinity) (all Miltenyi Biotec, Bergisch Gladbach, Germany) were used.

### Determination of CAR-T and CAR-NK cell functionality in coculture with adherent cells

U-87 MG and MeWo cells were stably transduced to express the octapeptide VLQSQRTD or the elongated peptide SGKALVLQSQRTD with an additional rigid linker and intracellular GFP, respectively, as well as a control GFP. 5 × 10^3^ stably transduced cells were then seeded per well in a 96-well flat bottom plate (Greiner, Kremsmünster, Austria) and incubated overnight. CAR-T cells were added in E:T ratios of 2.5:1 and 1:1, and CAR-NK cells were added in E:T ratios of 1:1 and 0.5:1. Annexin V NIR (Sartorius, Göttingen, Germany) was used at a final dilution of 1:800 to stain apoptotic cells. For analysis, the IncuCyte SX5 (Sartorius, Göttingen, Germany) was used. Phase, green, and NIR channel were recorded every 60 min in a 4x or 10x magnification.

### Viral infection with modified MV

1.5 × 10^4^ U-87 MG and MeWo cells were seeded 1 day prior to inoculation. MV suspensions were diluted in Opti-MEM at designated MOIs. For mock-infected controls, pure Opti-MEM was added. The inoculum was replaced by the respective culture medium after 2 h. Phase-contrast and fluorescence microscopy images were recorded using the IncuCyte SX5. After incubation of 48 h, cells were detached from 48-well plates using TrypLE Express without phenol red (Thermo Fisher Scientific, Waltham, MA, USA) and washed once. The cells were then stained with 500 ng of the 12-F7-C12-E3 antibody received from ProteoGenix for 30 min at 4°C. Then, a secondary staining using the Alexa Fluor 647-conjugated AffiniPure F(ab’)2 fragment goat anti-mouse IgG, F(ab’)2 fragment-specific antibody (Jackson ImmunoResearch, West Grove, PA, USA) was carried out. Tumor cells were then analyzed using flow cytometry for expression of GFP and the VLQSQRTD octapeptide.

### Determination of CAR-T and CAR-NK cell functionality in coculture with adherent tumor cells and MV

5 × 10^3^ U-87 MG or MeWo cells were seeded per well in a 96-well flat-bottom plate. After 24 h, MV was added at an MOI of 1 for delivery of VLQSQRTD and SGKALVLQSQRTD and an MOI of 0.1 for mock-GFP in 50 μL. After incubation at 37°C for 2 h, the inoculum was removed and replaced with DMEM containing 10% FCS. MOI for infection was determined based on GFP signal after 48 h of incubation. Forty-eight h after the infection, effector cells were added. Annexin V NIR (Sartorius, Göttingen, Germany) was used at a final dilution of 1:800 to stain apoptotic cells. For analysis, the IncuCyte SX5 (Sartorius, Göttingen, Germany) was used. Phase, green, and NIR channel were recorded every 60 or 120 min in a 4x magnification.

For detection of CAR-T and CAR-NK cell counts after the coculture, CAR-T cells were labeled with 1 μM CellTrace Violet and 1 μM CellTrace FarRed according to the manufacturer’s instructions, respectively (Thermo Fisher Scientific, Waltham, MA, USA).

For evaluation of CAR-T and CAR-NK cell activation following coculture with tumor cells and MV, staining with CD3 (CD3 antibody, VioBlue, anti-human, REAfinity), CD16 (CD16 antibody, anti-human, PerCP-Vio700, REAfinity), OX40 (CD134 antibody, PerCP-Vio700, anti-human, REAfinity), NKG2D (CD314 antibody, PE, anti-human, REAfinity), DNAM-1 (CD226 antibody, PE-Vio770, anti-human, REAfinity), 2B4 (CD244 antibody, APC, anti-human, REAfinity), CD48 (CD48 Antibody, APC-Vio770, anti-human, REAfinity) (all Miltenyi Biotec, Bergisch Gladbach, Germany), and OX40L (CD252 antibody, BV421, anti-human) (Becton Dickinson [BD], New Jersey, USA) was carried out after 72 h of coculture.

### IFN-γ ELISA

After an incubation time of 72 h, culture supernatants were collected and frozen immediately at −20°C for analysis of IFN-γ release via ELISA. Briefly, supernatants were diluted 1:2 with reagent diluent, and IFN-γ DuoSet ELISA (R&D Systems, Minneapolis, MN, USA) was conducted according to the manufacturer’s instructions.

### MACSPlex assay

For assessing cytokine and chemokine secretion from cocultures of target cells, CAR-NK, CAR-T, and OV, cell culture supernatants were harvested after 72 h. Supernatants were then analyzed using the MACSPlex cytokine T/NK cell kit, human (Miltenyi Biotec, Bergisch Gladbach, Germany) according to the manufacturer’s instructions on an MACS QuantX flow cytometer (Miltenyi Biotec, Bergisch Gladbach, Germany).

### Fluorescence microscopy

Microscopic analysis was performed using a Nikon Eclipse Ti-E microscope (Nikon, Tokio, Japan). Briefly, A375 cells were cultured in 6-well plates and stably transduced via puromycin selection as described earlier. EGFP was detected by using a blue laser and a 40× magnification.

### Statistical analysis

Data are presented as mean ± SEM. Statistical analysis was performed using GraphPad Prism 7 software (GraphPad, La Jolla, CA, USA). Two-way ANOVA followed by Dunnett’s multiple-comparisons test was used to compare experimental groups to one specific control group, unless otherwise stated. Two-way ANOVA with Tukey’s multiple-comparisons test was calculated for comparison of three or more experimental groups among each other.

## Data availability

The complete dataset supporting this study’s findings is presented within the manuscript. For access to the original raw data, interested parties may contact the corresponding author directly.

## Acknowledgments

This research was funded by the Fraunhofer Internal Programs under grant no. Attract 131-600004 and grant no. SME 40-08083, as well as the German Cancer Aid, Bonn, through the “CAR Factory” consortium.

## Author contributions

D.S. was responsible for funding acquisition, conceptualization, and supervision and oversaw project administration. A.R. was responsible for conceptualization and developed the methodology, led the investigation, curated the data, performed formal analysis and visualization, and wrote the original draft. C.E.E. was responsible for conceptualization and providing resources. M.S.C.F. and F.V.H. contributed resources. A.S., M.L., and N.D. aided in acquiring data. M.S. performed formal analysis. U.K. and S.F. were responsible for funding acquisition and contributed to the review and editing process.

## Declaration of interests

The authors declare no competing interests.

## Declaration of generative AI and AI-assisted technologies in the writing process

During the preparation of this work, the authors used DeepL, Perplexity, and ChatGPT to improve language and readability. After using these tools, the authors reviewed and edited the content as needed and take full responsibility for the content of the publication.

## Supplemental information

Document S1. Figures S1–S10

Document S2. Article plus supplemental information
