# Supplementary material for: Enhanced solid tumor cell targeting by a neoepitope-encoding oncolytic measles virus combined with CAR therapy
Source: Mol Ther Oncol. 2025 Aug 30;33(4):201043. doi: 10.1016/j.omton.2025.201043 (PMC12495166; doi:10.1016/j.omton.2025.201043)
Supplement: Document S1. Figures S1–S10 [file mmc1.pdf]

OMTON, Volume *33*

Supplemental information

Enhanced solid tumor cell targeting by a neoepitope-encoding oncolytic measles virus combined with CAR therapy

Alexander Renner, Maximiliane S.C. Finkbeiner, Ferdinand V. Haas, Anika Stahringer, Max Lindow, Nicolas Delaroque, Michael Szardenings, Stephan Fricke, Ulrike Koehl, Christine E. Engeland, and Dominik Schmiedel

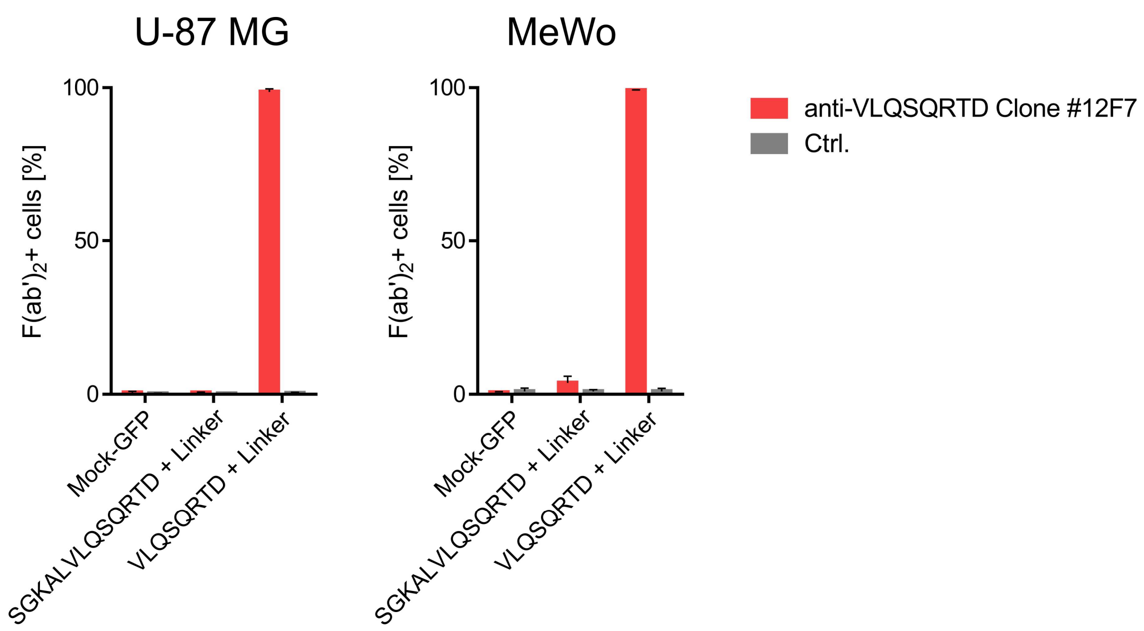

Figure S1: F(ab')$_2$ signal of stably transduced U-87 MG (left) and MeWo cells (right) expressing GFP only, the octapeptide VLQSQRTD or SGKALVLQSQRTD including a rigid $(EAAAK)_4A$ linker after incubation with antibody #12F7. Data is shown from n = 3 experiments as mean (SEM).

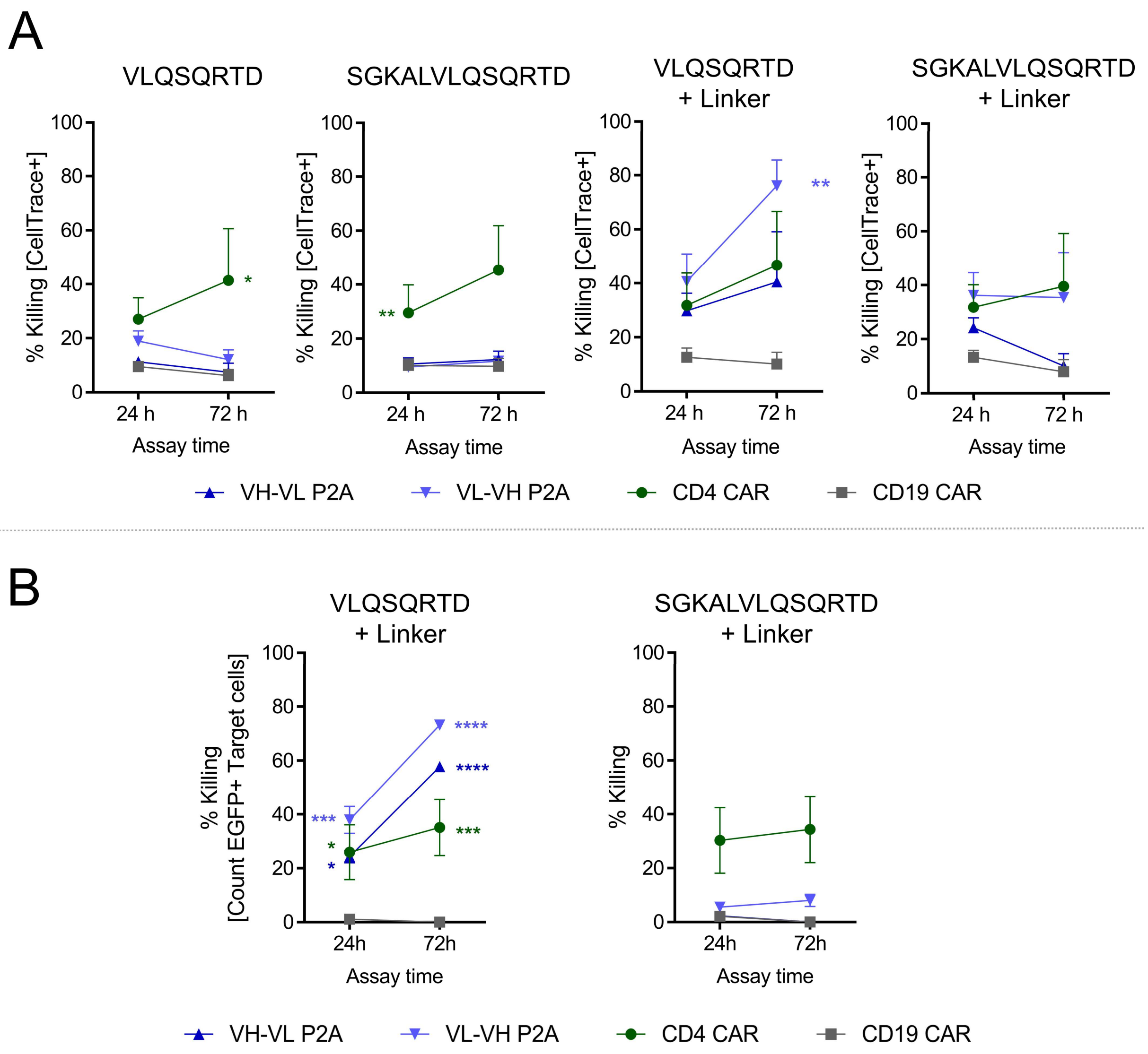

Figure S2: (A) CAR-T cells were cocultured with Mac-1 cells stably transduced to express indicated transgenes for 24 h or 72 h in an E:T ratio of 1:1. Tumor cells were labeled with CellTrace Violet and killing was assessed via PI staining. Killing is depicted as ratio of PI+ cells in cocultures and independently cultured Mac-1 cells. Data from n = 4 experiments is depicted as mean (SEM). (B) CAR-T cells were cocultured with T cells from the same donor transduced to express indicated transgenes for 24 h or 72 h in an E:T ratio of 1:1. T cells used as target cells were labeled with CellTrace Violet and killing was assessed via PI staining. Only EGFP+ target T cells were analyzed for PI staining. Killing is depicted as the ratio of PI+ cells in cocultures and independently cultured target T cells. Data from n = 4 independent experiments is shown as mean (SEM), * = $p \leq 0.05$, ** = $p \leq 0.01$, *** = $p \leq 0.001$, **** = $p \leq 0.0001$.

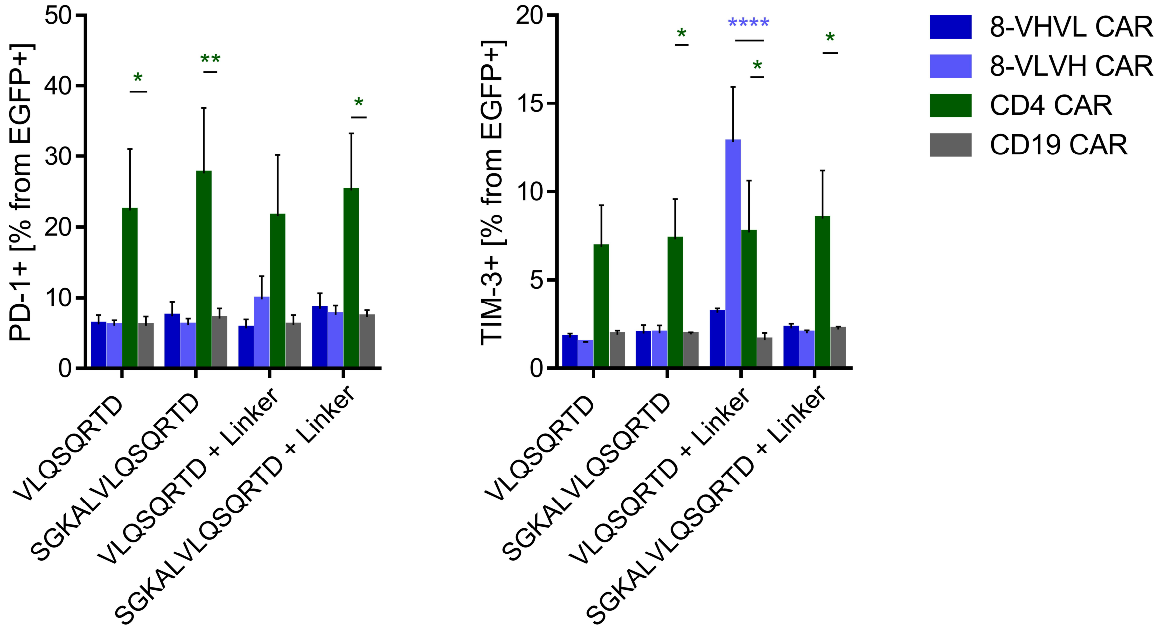

Figure S3: (A) PD-1 and (B) TIM-3 expression was measured on CAR-T cells after incubation with transduced T cells as target cells for 72 h. Data is shown from EGFP+ CAR-T cells from n = 4 experiments as mean (SEM). Statistical analysis was performed by Two-way ANOVA followed by Dunnett's multiple-comparisons test and experimental groups were compared to CD19 CAR; * = $p \leq 0.05$, ** = $p \leq 0.01$, *** = $p \leq 0.001$, **** = $p \leq 0.0001$.

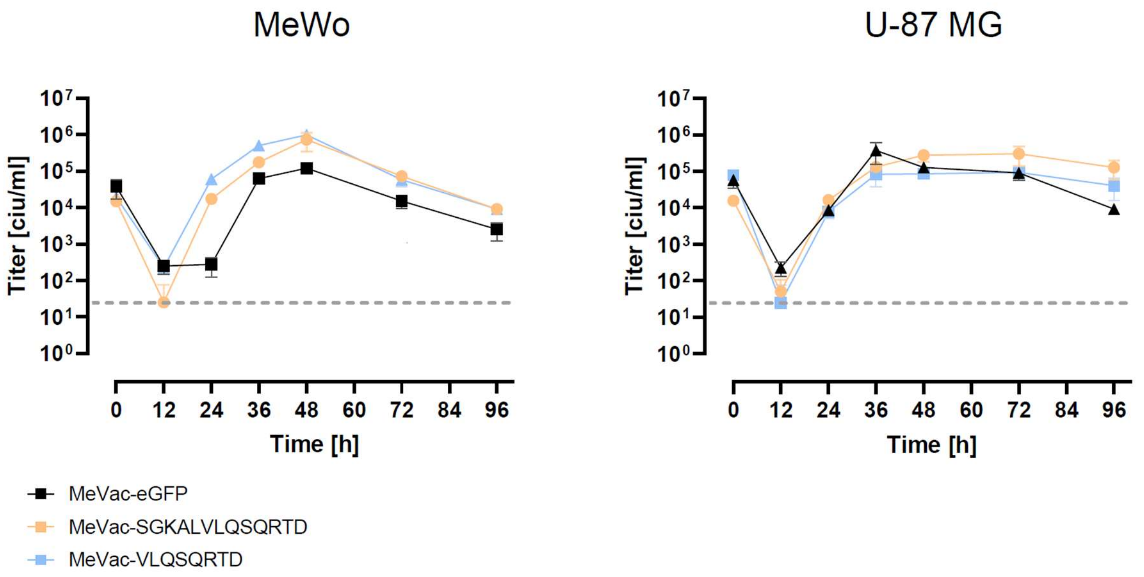

Figure S4: Replication kinetics of MeVac strains carrying an eGFP transgene, the SGKALVLQSQRTD- or VLQSQRTD-peptide fused to GFP. One-step growth curves were generated by infection of cells at MOI 3 and titration of viral progeny at designated timepoints. Grey dashed line marks the detection limit of the assay. One representative growth curve out of three biological replicates is shown as mean (SEM) from n = 4 technical replicates.

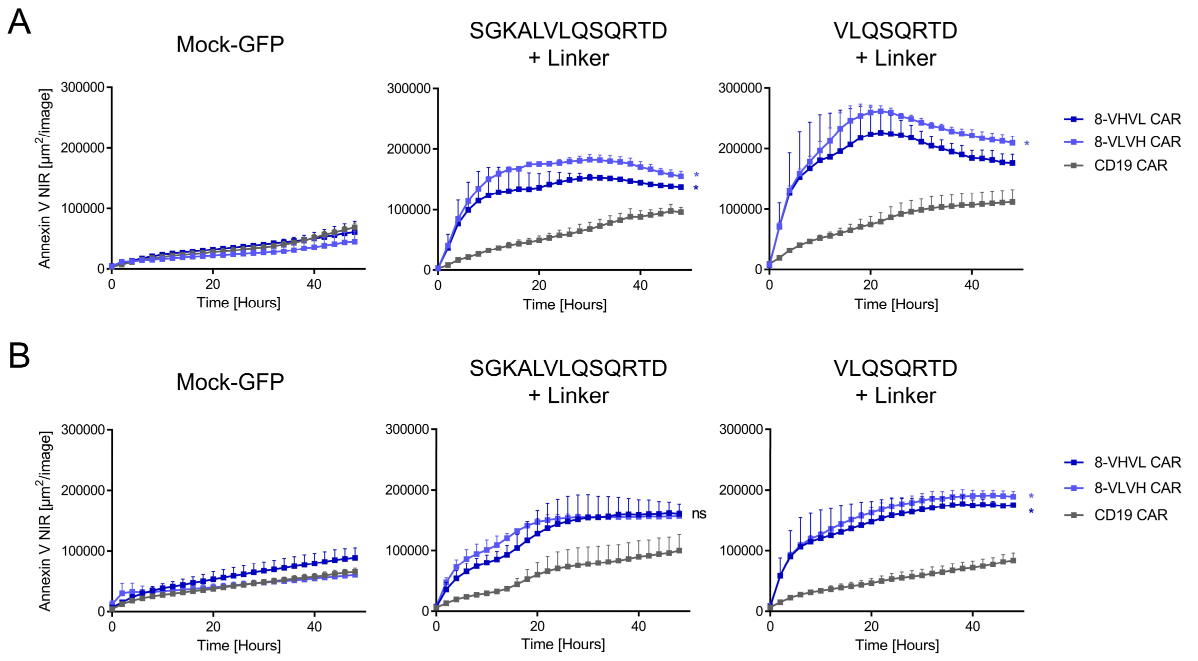

Figure S5: (A) CAR-T cells were cocultured with U-87 MG cells stably transduced to express GFP as well as SGKALVLQSQRTD or VLQSQRTD with a rigid linker. Annexin V NIR dye was used to distinguish apoptotic cells. Data from n = 2 independent experiments is shown as mean (SEM). (B) CAR-T cells were cocultured with MeWo cells stably transduced to express GFP as well as SGKALVLQSQRTD or VLQSQRTD with a rigid linker. Annexin V NIR dye was used to distinguish apoptotic cells. Data from n = 2 independent experiments is shown as mean (SEM). Statistical analysis was performed by Two-way repeated measures ANOVA with Tukey's multiple comparisons test, * = $p \leq 0.05$.

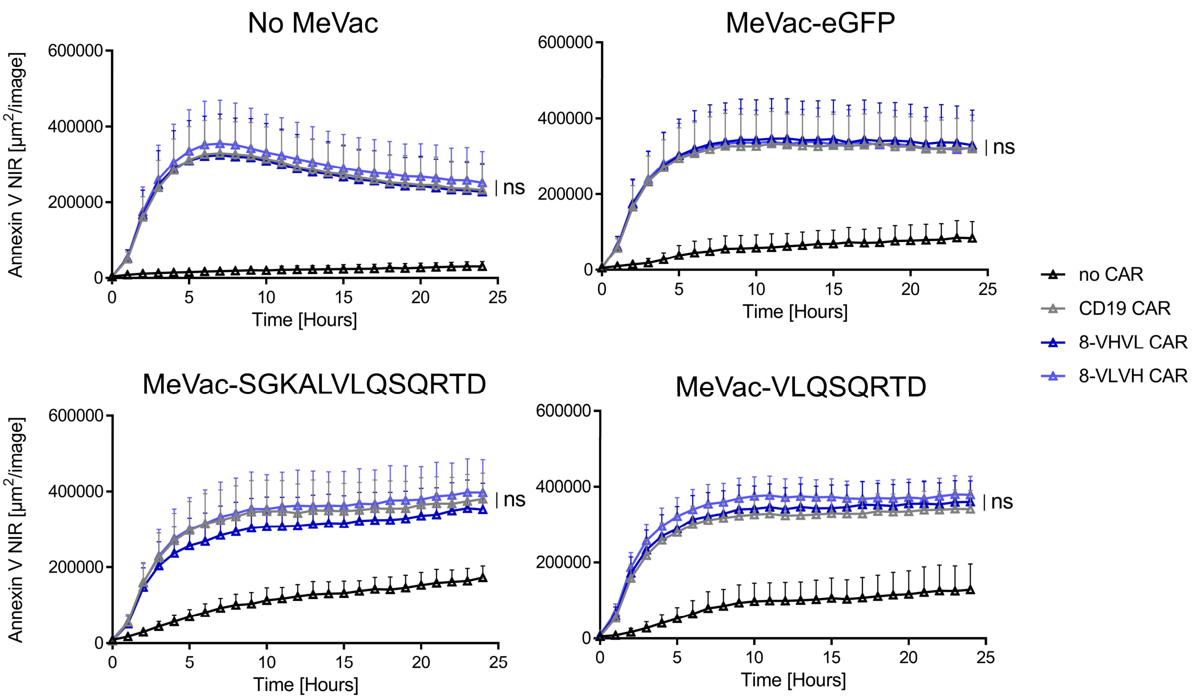

Figure S6: MeWo cells were inoculated with MeVac-eGFP, MeVac-SGKALVLQSQRTD or MeVac-VLQSQRTD. Forty-eight hours after inoculation, CAR-NK cells were added and apoptosis was monitored by addition of Annexin V NIR dye to the culture medium. Data from n = 4 independent experiments are shown as mean (SEM). Statistical analysis was performed by Two-way repeated measures ANOVA with Tukey's multiple comparisons test.

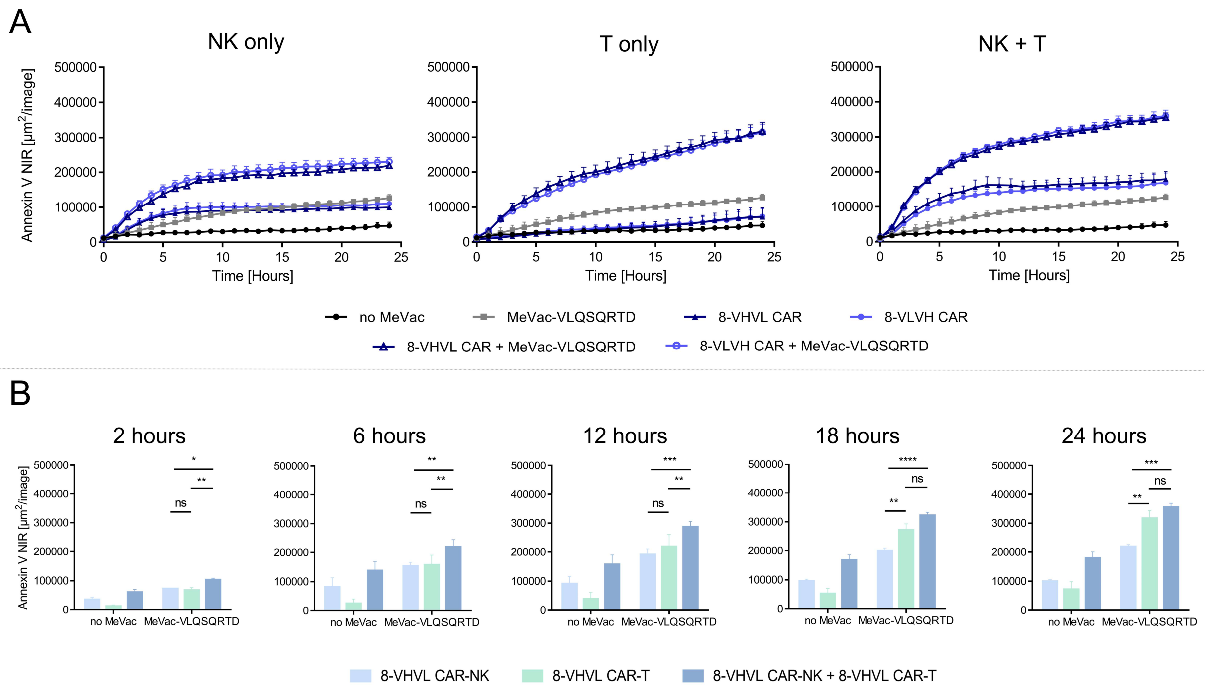

Figure S7: (A) Killing of MeWo cells inoculated with MeVac-VLQSQRTD and then cocultured with 8-VHVL and 8-VLVH CAR-NK, CAR-T or both CAR-NK and CAR-T cells is shown. Annexin V NIR dye was used to identify apoptotic cells. Data is shown as area per image and is depicted from n = 4 independent experiments as mean (SEM). (B) Annexin V NIR signals from plots in A are shown at five different timepoints over the course of the experiment as mean (SEM). Statistical analysis in B was performed by Two-way ANOVA with Tukey's multiple comparisons test, * = $p \leq 0.05$, ** = $p \leq 0.01$, *** = $p \leq 0.001$, **** = $p \leq 0.0001$.

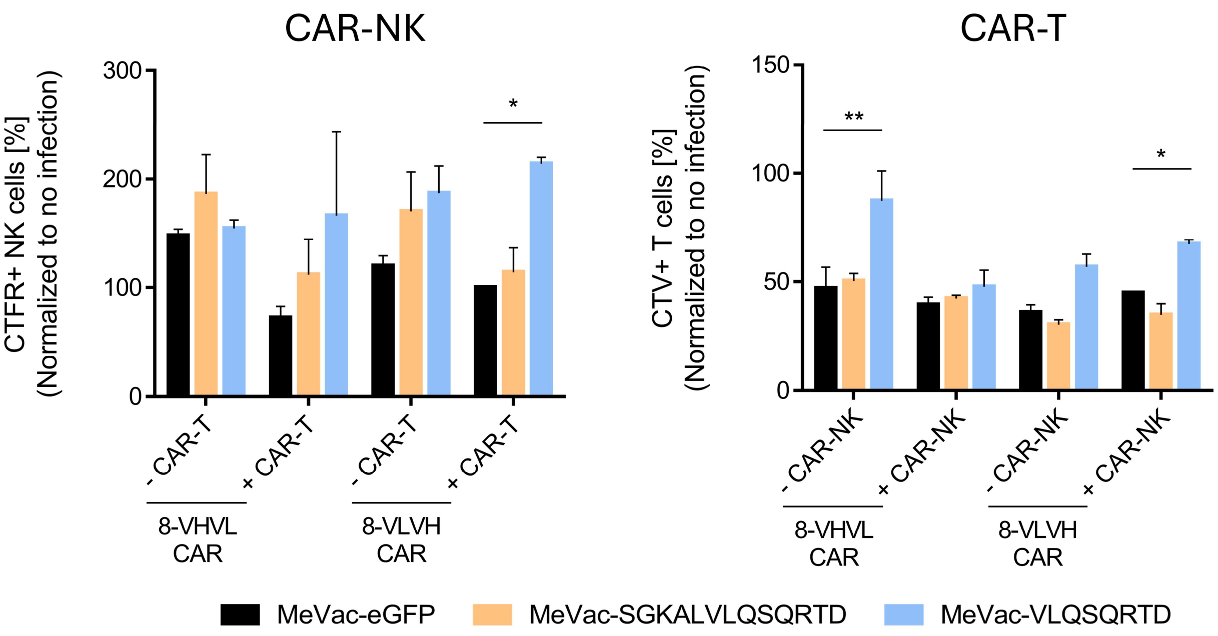

Figure S8: Detected cell counts of CellTrace FarRed+ CAR-NK cells in coculture with MeWo in absence or presence of CAR-T cells (left plot) and CellTrace Violet+ CAR-T cells in coculture with MeWo in absence or presence of CAR-NK cells (right plot) normalized to cell counts of samples without MeVac infection are shown. Values are shown as mean (SEM) from n = 2 independent experiments, * = p ≤ 0.05, ** = p ≤ 0.01

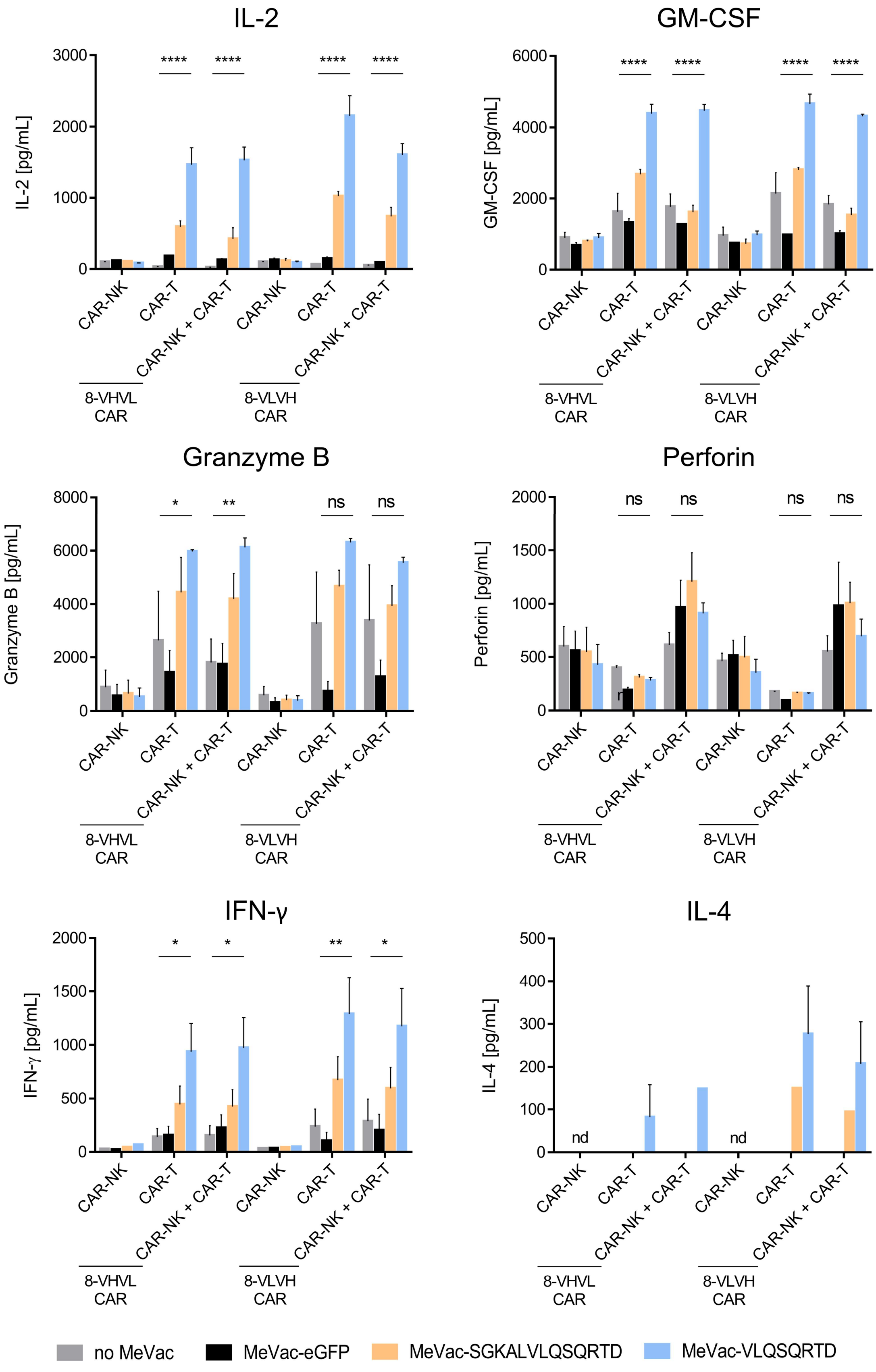
IL-2
IL-2 [pg/mL]
GM-CSF
GM-CSF [pg/mL]
Granzyme B
Granzyme B [pg/mL]
Perforin
Perforin [pg/mL]
IFN-γ
IFN-γ [pg/mL]
IL-4
IL-4 [pg/mL]
nd
****
*
**
ns
CAR-NK
CAR-T
CAR-NK + CAR-T
8-VHVL CAR
8-VLVH CAR
no MeVac
MeVac-eGFP
MeVac-SGKALVLQSQRTD
MeVac-VLQSQRTD

Figure S9: U-87 MG cells were inoculated with MeVac and then cocultured with 8-VHVL and 8-VLVH CAR-NK, CAR-T or both CAR-NK and CAR-T cells. After 72 h, supernatant was harvested and cytokine secretion was quantified. Data is shown as mean (SEM) from n = 2 independent experiments. Statistical analysis of was performed by Two-way ANOVA with Tukey's multiple comparisons test, * = $p \leq 0.05$, ** = $p \leq 0.01$, *** = $p \leq 0.001$, **** = $p \leq 0.0001$.

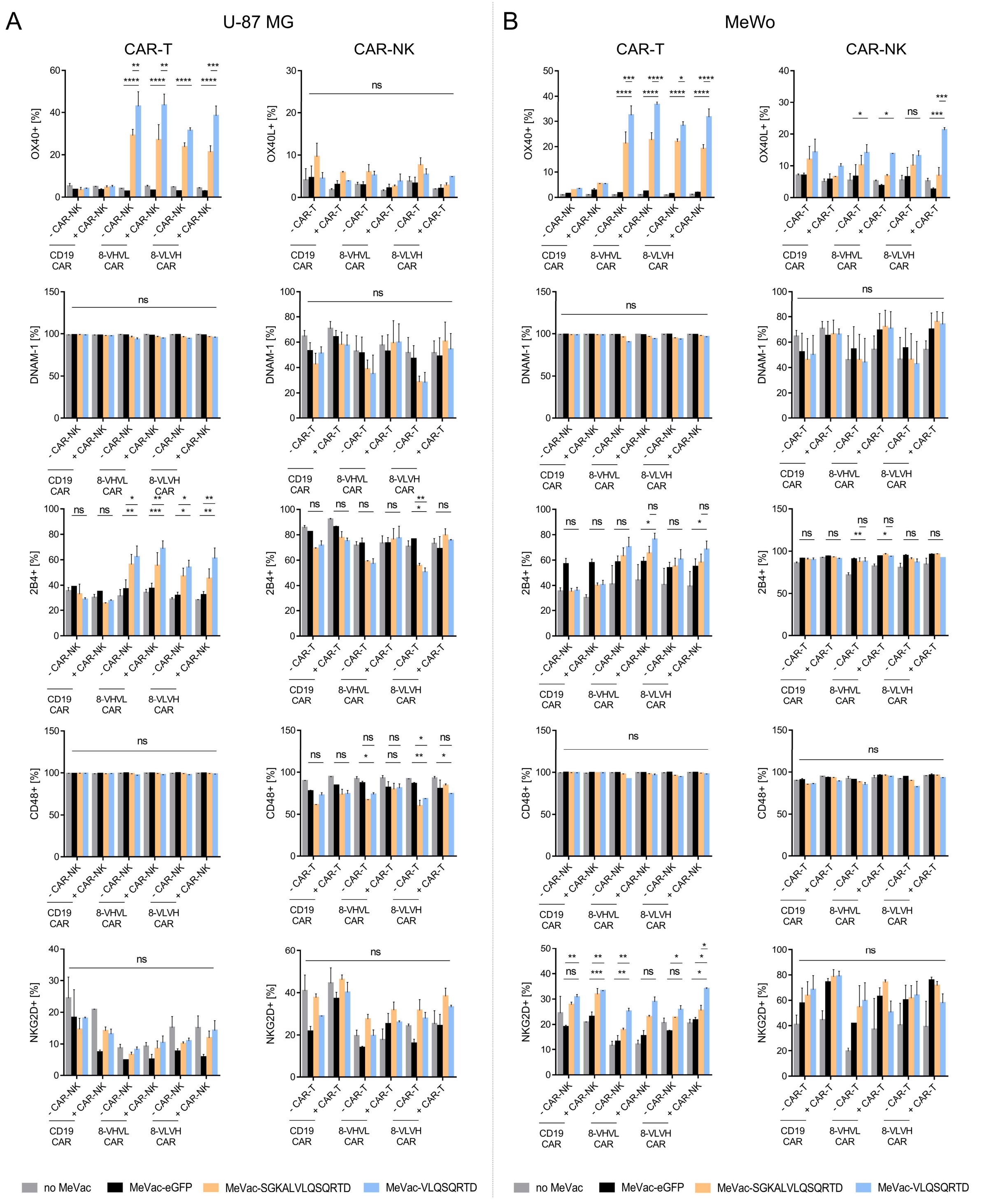

Figure S10: Expression of a set of receptors and ligands was measured on CAR-T and CAR-NK cells after coculture of 72 h with U-87 MG (A) and MeWo (B) inoculated with MeVac in the presence or absence of CAR-NK or CAR-T cells, respectively. Data is shown from EGFP+ CAR-T and CAR-NK cells from n = 2 experiments as mean (SEM). Statistical analysis was performed by Two-way ANOVA followed by Dunnett's multiple-comparisons test and

experimental groups were compared to no MeVac; * = $p \leq 0.05$, ** = $p \leq 0.01$, *** = $p \leq 0.001$, **** = $p \leq 0.0001$.
